# Genome-Wide Analysis Reveals Selection for Important Traits in Domestic Horse Breeds

**DOI:** 10.1371/journal.pgen.1003211

**Published:** 2013-01-17

**Authors:** Jessica L. Petersen, James R. Mickelson, Aaron K. Rendahl, Stephanie J. Valberg, Lisa S. Andersson, Jeanette Axelsson, Ernie Bailey, Danika Bannasch, Matthew M. Binns, Alexandre S. Borges, Pieter Brama, Artur da Câmara Machado, Stefano Capomaccio, Katia Cappelli, E. Gus Cothran, Ottmar Distl, Laura Fox-Clipsham, Kathryn T. Graves, Gérard Guérin, Bianca Haase, Telhisa Hasegawa, Karin Hemmann, Emmeline W. Hill, Tosso Leeb, Gabriella Lindgren, Hannes Lohi, Maria Susana Lopes, Beatrice A. McGivney, Sofia Mikko, Nicholas Orr, M. Cecilia T. Penedo, Richard J. Piercy, Marja Raekallio, Stefan Rieder, Knut H. Røed, June Swinburne, Teruaki Tozaki, Mark Vaudin, Claire M. Wade, Molly E. McCue

**Affiliations:** 1 College of Veterinary Medicine, University of Minnesota, St. Paul, Minnesota, United States of America; 2 School of Statistics, University of Minnesota, Minneapolis, Minnesota, United States of America; 3 Department of Animal Breeding and Genetics, Swedish University of Agricultural Sciences, Uppsala, Sweden; 4 Department of Veterinary Science, University of Kentucky, Lexington, Kentucky, United States of America; 5 School of Veterinary Medicine, University of California Davis, Davis, California, United States of America; 6 Equine Analysis, Midway, Kentucky, United States of America; 7 Department of Veterinary Clinical Science, University Estadual Paulista, Botucatu, Brazil; 8 School of Veterinary Medicine, University College Dublin, Dublin, Ireland; 9 Department of Agriculture, University of the Azores, Angra do Heroísmo, Portugal; 10 Faculty of Veterinary Medicine, University of Perugia, Perugia, Italy; 11 College of Veterinary Medicine and Biomedical Science, Texas A&M University, College Station, Texas, United States of America; 12 Institute for Animal Breeding and Genetics, University of Veterinary Medicine Hannover, Hannover, Germany; 13 Animal Health Trust, Lanwades Park, Newmarket, Suffolk, United Kingdom; 14 Animal Genetics and Integrative Biology Unit, French National Institute for Agricultural Research, Jouy en Josas, France; 15 Faculty of Veterinary Science, University of Sydney, New South Wales, Australia; 16 Nihon Bioresource College, Koga, Ibaraki, Japan; 17 Faculty of Veterinary Medicine, University of Helsinki, Helsinki, Finland; 18 College of Agriculture, Food Science and Veterinary Medicine, University College Dublin, Dublin, Ireland; 19 Institute of Genetics, University of Bern, Bern, Switzerland; 20 Breakthrough Breast Cancer Research Centre, Institute of Cancer Research, London, United Kingdom; 21 Comparative Neuromuscular Diseases Laboratory, Royal Veterinary College, London, United Kingdom; 22 Swiss National Stud Farm SNSTF, Agroscope Liebefeld-Posieux Research Station, Avenches, Switzerland; 23 Department of Basic Sciences and Aquatic Medicine, Norwegian School of Veterinary Science, Oslo, Norway; 24 Animal DNA Diagnostics, Cambridge, United Kingdom; 25 Department of Molecular Genetics, Laboratory of Racing Chemistry, Utsunomiya, Tochigi, Japan; University of Washington, United States of America

## Abstract

Intense selective pressures applied over short evolutionary time have resulted in homogeneity within, but substantial variation among, horse breeds. Utilizing this population structure, 744 individuals from 33 breeds, and a 54,000 SNP genotyping array, breed-specific targets of selection were identified using an F_ST_-based statistic calculated in 500-kb windows across the genome. A 5.5-Mb region of ECA18, in which the myostatin *(MSTN)* gene was centered, contained the highest signature of selection in both the Paint and Quarter Horse. Gene sequencing and histological analysis of gluteal muscle biopsies showed a promoter variant and intronic SNP of *MSTN* were each significantly associated with higher Type 2B and lower Type 1 muscle fiber proportions in the Quarter Horse, demonstrating a functional consequence of selection at this locus. Signatures of selection on ECA23 in all gaited breeds in the sample led to the identification of a shared, 186-kb haplotype including two doublesex related mab transcription factor genes (*DMRT2* and *3*). The recent identification of a *DMRT3* mutation within this haplotype, which appears necessary for the ability to perform alternative gaits, provides further evidence for selection at this locus. Finally, putative loci for the determination of size were identified in the draft breeds and the Miniature horse on ECA11, as well as when signatures of selection surrounding candidate genes at other loci were examined. This work provides further evidence of the importance of *MSTN* in racing breeds, provides strong evidence for selection upon gait and size, and illustrates the potential for population-based techniques to find genomic regions driving important phenotypes in the modern horse.

## Author Summary

A breed of the horse typically consists of individuals sharing very similar aesthetic and performance traits. However, a great deal of variation in traits exists between breeds. The range of variation observed among breeds can be illustrated by the size difference between the Miniature horse (0.74 m and 100 kg) and draft horse (1.8 m and 900 kg), or by comparing the optimum racing distance of the Quarter Horse (1/4 mile) to that of the Arabian (100 miles or more). In this study, we exploited the breed structure of the horse to identify regions of the genome that are significantly different between breeds and therefore may harbor genes and genetic variants targeted by selective breeding. This work resulted in the identification of variants in the Paint and Quarter Horse significantly associated with altered muscle fiber type proportions favorable for increased sprinting ability. A strong signature of selection was also identified in breeds that perform alternative gaits, and several genomic regions identified are hypothesized to be involved in the determination of size. This study has demonstrated the utility of this approach for studying the equine genome and is the first to show a functional consequence of selective breeding in the horse.

## Introduction

Since domestication of the horse approximately 5,000 years ago [1]–[3], selective breeding has been directed mainly toward the use of the horse in agriculture, transportation, and warfare. Within the past 400 years, the founding of formal breed registries and continued breed specialization has focused more upon preserving and improving traits related to aesthetics and performance. As a result, most horse breeds today are closed populations with high phenotypic and genetic uniformity of individuals within the breed, but with a great deal of variation among breeds. High-throughput, whole-genome SNP arrays can now be used to exploit this population structure to identify the effects of selection upon the equine genome. Once genomic regions targeted by selection are detected, the variants and processes that have contributed to desired phenotypes within breeds and across performance groups can be more readily identified.

Population-based approaches to identify signals of selection using loss of heterozygosity and/or other diversity indices have been successful in several domestic species. In dogs, these studies have led to the identification of genomic regions implicated in the selection of characteristics such as coat color and texture, body size, skin wrinkling, and disease [4], [5], as well as the identification of signals of selection across genes with both known and unknown function [6]–[9]. Similar studies in cattle have identified regions of interest that encompass genes with known or potential importance for muscling, feed efficiency, milk production, and reproduction [10]–[13], and genomic targets of selection for reproductive traits, coat pigmentation, and lack of horns (polled) have been identified in the sheep [14]. While a considerable number of traits are under selection in the many breeds and performance groups of the horse, the only prior population-based study of selection in the horse utilized microsatellite loci to identify loci of importance to the Thoroughbred with respect to three other breeds [15].

This report, using an autosomal single nucleotide polymorphism (SNP) array and a sample set of 33 different breeds, represents the first large-scale study of how selective pressures have shaped the equine genome. Within each breed, genotypes in 500 kb windows were evaluated to identify those more divergent from the other breeds in the study than expected, therefore signifying potential genomic targets of selection. Of the regions of putative selection identified, priority was given to regions with the highest *d_i_* value within a breed, regions with consecutive windows of significance covering at least 1 Mb, regions shared by breeds with similar phenotype, or regions showing significance near genes with known or suggested functional effect. Regions chosen for follow-up studies were further investigated by phasing genotypes to discover any extended haplotypes present at high frequency in the breed(s) of interest. Haplotypes were then scanned to identify candidate genes for follow-up study. This report focuses upon regions of selection that are hypothesized to be involved in coat color, performance, gait (pattern of locomotion) and size. Our results support the use of this technique in the horse to identify novel variants of functional importance and to further elucidate how selection has shaped the equine genome.

## Results

### Overview

A total of 744 horses representing 33 breeds (average 22.5 horses/breed) genotyped with the Equine SNP50 Beadchip (Illumina) were included in the analysis. Information regarding the breeds, sample sizes and breed-specific phenotypes is found in Table 1. Horses were selected to represent a random sample of the breed whenever possible and were chosen to be unrelated to one another at, or more recent to, the grandsire/dam level. In the case where pedigree information was not available, horses were removed from the analysis so no pair had a genome sharing value of greater than 0.3 (see methods). All horses included in the study genotyped at a rate greater than 0.98.

**Table 1:** Breeds included in the study, sample size, and distinguishing phenotype(s).

Breed	N	Breed-defining Trait(s)
Akhal Teke	19	endurance riding
Andalusian	18	sport horse
Arabian	24	endurance riding
Belgian	30	draft
Caspian Pony	18	small size
Clydesdale	24	draft
Exmoor	24	small size; hardiness
Fell Pony	21	small size
Finnhorse	27	race (trot); light draft
Franches-Montagnes	19	light draft
French Trotter	17	race (trot)
Hanoverian	15	sport horse
Icelandic	25	gait: 4-beat lateral amble, pace
Mangalarga Paulista	15	riding horse
Miniature	21	extreme small size
Mongolian	19	meat, milk, riding
Morgan	40	riding, driving
New Forest Pony	15	riding horse
North Swedish Horse	19	draft
Norwegian Fjord	21	dun coat color; riding, driving
Paint	25	white patterned coat color; ranch work
Percheron	23	draft
Peruvian Paso	21	gait: 4-beat lateral amble
Puerto Rican Paso Fino	20	gait: 4-beat lateral amble
Quarter Horse	40	race (sprint); heavy muscling; ranch work
Saddlebred	25	gait (some): 4-beat amble, pace or fox trot
Shetland	27	small size
Shire	23	draft
Standardbred	25	race (trot)
Swiss Warmblood	14	sport horse
Tennessee Walking Horse	19	gait: 4-beat lateral amble
Thoroughbred	36	race (∼1 mile)
Tuva	15	metabolic efficiency; hardiness

The F_ST_-based statistic, *d_i_* [4], was calculated for autosomal SNPs in 500 kb windows, with a minimum of 4 SNPs per window, and defining the populations by breed. The *d_i_* statistic is a summation at each window of pairwise F_ST_ values for each breed combination, corrected by the value expected from genome-wide calculations; therefore, a large value of *d_i_* indicates greater divergence at that 500 kb window than that observed across the genome as a whole. In total, 23,401 SNPs were evaluated within 3,229 windows (68.7% of the autosomes), averaging 7.25 SNPs per window (range 4–20). SNPs from the full data set not included in the analysis of *d_i_* were largely removed due to failure to meet the minimal SNP density requirement. The 33 windows within each breed, which fell into the upper 99^th^ percentile of the empirical distribution, were considered putative signatures of selection; these regions in each breed are listed in Table S1. The maximum *d_i_* value per breed ranged from 33.8 in the New Forest Pony to 104.4 in the Peruvian Paso. Of the 3,229 windows analyzed, 695 (2.7%) were significant (within the upper 99^th^ percentile) in at least one breed. The *d_i_* plots from all breeds in the study are provided in Figure S1.

### Utility of the Statistic—Identification of Common Haplotypes Containing Known Coat Color Loci

Prior work has suggested that coat color was a target of selection early in horse domestication [2] and several breeds continue to be selected for a uniform coat color or color patterns. Two examples of selection for coat color include the light chestnut coat desired in Belgian draft horses bred in the United States, and the dun coloration characteristic of the Norwegian Fjord.

Chestnut coat color is the result of a recessive mutation of the melanocortin 1 receptor (*MC1R*) [16]. This *MC1R* variant represents a gene with known effect that has been shown to be contained within an extended haplotype [17]. Genotypes for the *MC1R* locus were available for most horses in 14 of the breeds as reported previously [17]. In this sample, two breeds, the Morgan and Belgian, were fixed for the missense mutation that results in the base coat color of chestnut.

**Figure 1:**
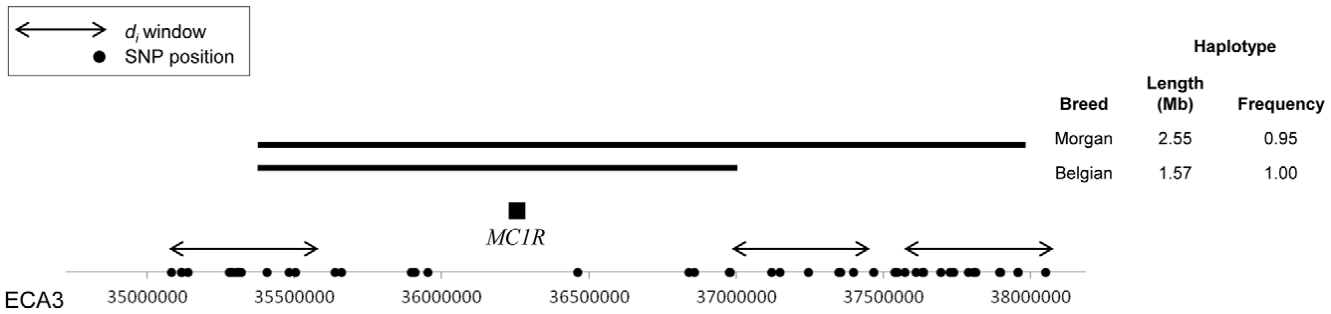
Haplotypes surrounding the *MC1R* locus in the Morgan and Belgian. Extended haplotypes surrounding the *MC1R* locus on ECA3 in Morgan and Belgian horses. SNPs on the Equine SNP50 Beadchip are designated on the x-axis as dots and windows where *d_i_* was calculated are shown with double-sided arrows. The common haplotype is shown as a solid, horizontal bar. As the result of haplotype length in each breed, the *d_i_* statistic identified the signature of selection around *MC1R* in the Morgan, but not in the Belgian due to poor polymorphic SNP coverage resulting in no calculation of *d_i_* over this region.

Due to SNP density, *d_i_* was not calculated across the *MC1R* locus itself. However, the window with the highest *d_i_* value in the Morgan was a region on ECA3 with two consecutive significant windows (ECA3:37,825,015; *d_i_* = 67.22) near the *MC1R* locus (ECA3:36,259,276–36,260,354) (Figure S1). Phasing of the genotypes uncovered a 1.57 Mb, 12 SNP haplotype spanning *MC1R* in all Morgan chromosomes (N = 80); this haplotype extended 2.55 Mb in 95% of the chromosomes (Figure 1). Although the Belgian population had the identical 1.57 Mb haplotype across the *MC1R* locus, it did not have a high-frequency haplotype extending into the regions in which *d_i_* was calculated. As the result of the different haplotype lengths in each breed, the *d_i_* statistic identified the signature of selection around *MC1R* in the Morgan, but not in the Belgian (Figure 1 and Figure S1).

The frequency of the mutant *MC1R* allele in the 12 other breeds for which the *MC1R* genotype was known ranged from 0.19 in the Andalusian to 0.88 in the American Saddlebred (hereafter “Saddlebred”) (data not shown). In addition to the Morgan, significant *d_i_* values adjacent to the *MC1R* locus were found in the Finnhorse and Saddlebred, which each had a high frequency (0.85) of the same extended haplotype (2.19 and 2.64 Mb, respectively) across *MC1R* as that found in the Morgan (data not shown). Significant *d_i_* windows upstream and adjacent to the *MC1R* region were also found in the Andalusian, Exmoor Pony, Fell Pony, Icelandic, North Swedish Horse, and Shire (Figure S1); however, these populations did not have a high frequency of the *MC1R* haplotype consistent with chestnut coat color.

Another instance of detection of a coat color locus was found on ECA8 in the Norwegian Fjord, a breed selected for the dun coat color dilution. A significant window on ECA8, centered at 17.5 Mb, is in the same region as the genetically mapped locus associated with the dun dilution [18].

### ECA18—Muscle Characteristics and Racing Aptitude

Racing performance is a trait of high economic importance to the equine industry. Variation in racing aptitudes range from that of the American Quarter Horse (hereafter “Quarter Horse”), which was originally bred to sprint ¼ mile (400 m), to the opposite extreme of the Arabian and Akhal Teke breeds that compete in endurance races up to and over 100 miles (160.9 km). Intermediate to the Quarter Horse and endurance horses, the Thoroughbred races distances ranging from 5/8 to 2 miles (1–3.2 km), and the Standardbred competes at a distance of approximately one mile (1600 m) under harness at a trot or pace rather than a gallop.

**Figure 2:**
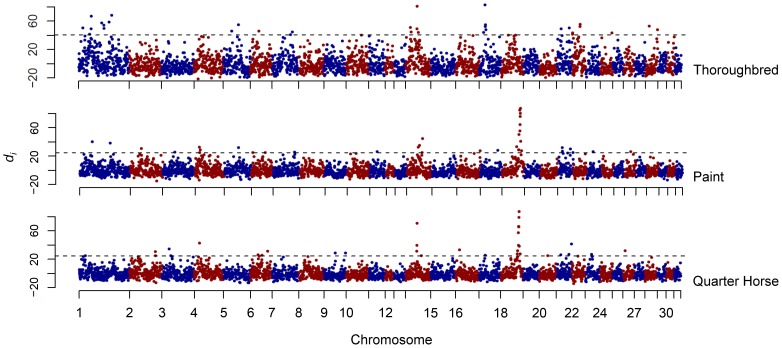
Genome-wide *d_i_* values for the Thoroughbred, Paint, and Quarter Horse. Output of the *d_i_* calculation for the Thoroughbred, Paint, and Quarter Horse. The *d_i_* value is plotted on the y axis and each autosome is shown in the x axis in alternating colors. Each dot represents one 500 kb window. The dashed horizontal line represents the 99^th^ percentile of the empirical distribution of *d_i_* for each breed.

A large region of putative selection was found on ECA18 in the American Paint Horse (hereafter “Paint”) and Quarter Horse populations, which included the highest *d_i_* values observed in each breed (Figure 2). Not only was the highest *d_i_* value for each breed found in this region, but 9 and 10 of the 11 consecutive windows (from 60.59 to 68.84 Mb), were significant in the Quarter Horse and Paint, respectively.

#### Haplotype analysis

**Figure 3:**
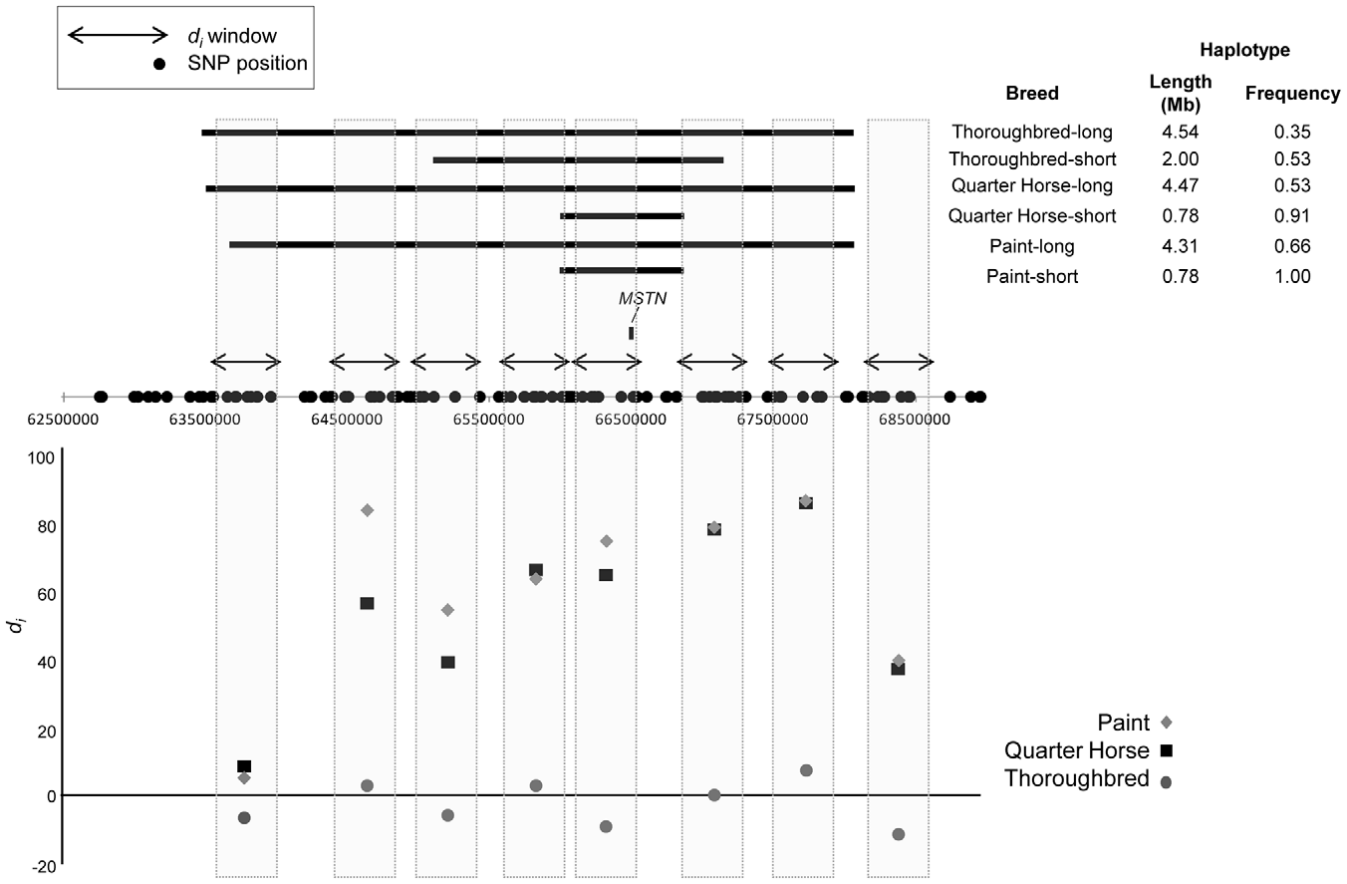
Significant signature of selection and associated haplotypes on ECA18 surrounding *MSTN*. (top) Haplotype size, position, and frequency for the minimal, shared and extended haplotypes (solid, horizontal bars) on ECA18 in the Thoroughbred, Quarter Horse, and Paint. Significant *d_i_* windows are shown as double-sided arrows. The position of the *MSTN* gene is shown. The identities of all genes found within the 0.78 Mb haplotype are given in Table S2. (bottom) *d_i_* values for each window in the region of *MSTN* for the three breeds.

Phasing of the genotypes across ECA18 showed a common, 780.7 kb haplotype composed of 21 SNPs, found in 91.3 and 100% of the Quarter Horse and Paint chromosomes, respectively. The same 780.7 kb haplotype, contained within a larger, 2 Mb haplotype, was also present in 52.8% of the Thoroughbred chromosomes (Figure 3). This 780.7 kb haplotype was present at a frequency of 0.04 across all other samples in the study.

#### Candidate gene sequencing

The myostatin gene (*MSTN*; ECA18: 66,490,208–66,495,180) was centered among 12 predicted or annotated genes in the 780.7 kb haplotype observed in the Paint and Quarter Horse (Table S2). Sanger DNA sequencing of *MSTN* was performed on 14 horses (8 Quarter Horse, 6 Thoroughbred) representing individuals with and without the 780.7 kb haplotype. A total of 19 variants were identified relative to the reference genome including a SINE insertion in the promoter region, which was also found in the Thoroughbred [19], as well as a SNP in intron 1 (g.66493737C/T) suggested to be predictive of optimal racing distance in Thoroughbreds [19], [20]. All SNPs identified in these 14 horses through sequence analysis of *MSTN* are presented in Table S3.

#### Association of *MSTN* variants with the common haplotype

To evaluate the frequency of the SINE insertion and association with the extended haplotype in an alternative sample, the SINE insertion was genotyped in 132 horses from which full-genome SNP data was available (121 Quarter Horses and 11 Paints). The 780.7 kb SNP haplotype was observed in 90.9% (240/264) of the chromosomes assayed and the SINE insertion was present in 95.8% (230) of the 240 instances in which the extended haplotype was observed. The intron 1 SNP was genotyped in 61 horses in which full-genome SNP data was available. Similar to the SINE, the intron 1 “C” allele was found in 96.9% of cases (94/97) in which the extended haplotype was present.

In this sample of Paint and Quarter Horses, the presence of the SINE was predictive of the “C” allele of the intron 1 SNP in 89.8% of the instances where data from both variants was available. Further, both the SINE and SNP were significantly associated with the extended haplotype shared among the Quarter Horse, Paint, and Thoroughbred (p<0.001 for each variant). The promoter SNP (g.66495715T/C) discussed in [21] and suggested to be associated with body composition (light vs. heavy breeds), was found in 8.7% of 23 Quarter Horses assayed and was not significantly associated with the extended haplotype, the SINE insertion, or intron 1 SNP (data not shown); no other *MSTN* variants were significantly associated with the haplotype in question.

#### Frequency of *MSTN* variants

In addition to the 132 horses above, 257 other individuals from whom full-genome SNP data was not available were genotyped for the SINE insertion. Of the 389 horses genotyped in total (357 Quarter Horse, 32 Paint), 309 (79.4%) were homozygous for the insert, 51 (13.1%) heterozygous, and 29 (7.5%) homozygous for the allele without the insert. In this sample, which largely represents horses collected for other laboratory studies and is not necessarily a random representation of each breed, the SINE was present at a frequency of 0.86. In a similar sample of 112 horses genotyped for the intron 1 SNP, 61 horses (54.5%) were homozygous for the “C” allele, 40 (35.7%) heterozygous, and 11 (9.8%) homozygous for the “T” allele; the frequency of the “C” allele of the intron 1 SNP in this sample was 0.88.

#### Gluteal muscle fiber typing

Because *MSTN* is a negative regulator of muscle development and a loss of function is known to influence muscle mass [22]–[26], we hypothesized that the SINE insertion may function to influence muscle fiber composition due to its position in the promoter region of the gene. To test this hypothesis, muscle fiber type proportions and diameters were quantified from gluteal muscle biopsies obtained from a standardized site [27] (see methods) of 79 Quarter Horses representing each genotype with respect to the SINE insertion (18 homozygous without the insert (NN), 31 heterozygous for the SINE insertion (NS), and 30 homozygous for the SINE insertion (SS)). Genotyping of the linked intron 1 SNP found that 34 of these horses were homozygous for the “C” allele, 33 heterozygous, and 9 homozygous for the wild-type, “T,” allele (3 were missing data).

The *MSTN* SINE insertion and intron 1 SNP were tested for significant association with fiber type proportions and diameter using multivariate analysis of variance (MANOVA). Age and gender have been shown to influence muscle fiber characteristics [28]–[31] and were therefore included as covariates in the analyses. An additive mode of inheritance was assumed.

**Figure 4:**
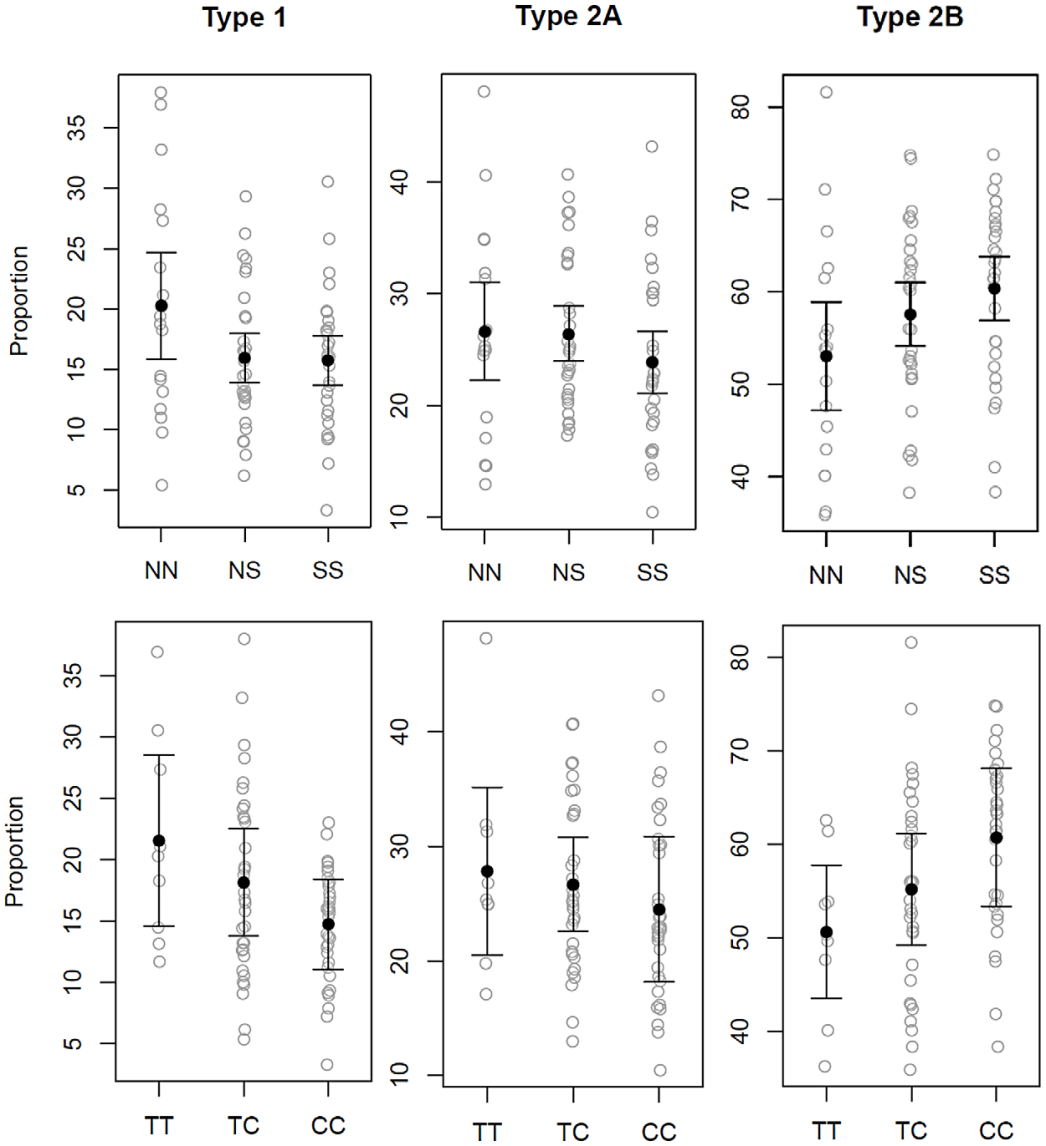
Gluteal fiber type proportions observed based upon *MSTN* SINE and intron 1 SNP genotype. Gluteal muscle fiber type proportions in 79 Quarter Horses based upon myostatin genotypes, not accounting for age and sex. The top panel shows the SINE (N = wild-type allele, S = SINE insertion) and bottom panel the intron 1 SNP genotypes. Each grey circle indicates fiber type proportion in one horse (y-axis). The mean is shown with a black dot and the error bars represent 95% confidence intervals around the mean. The SINE and SNP are each significantly associated with a lower proportion of Type 1 and higher proportion of Type 2B muscle fibers.

**Figure 5:**
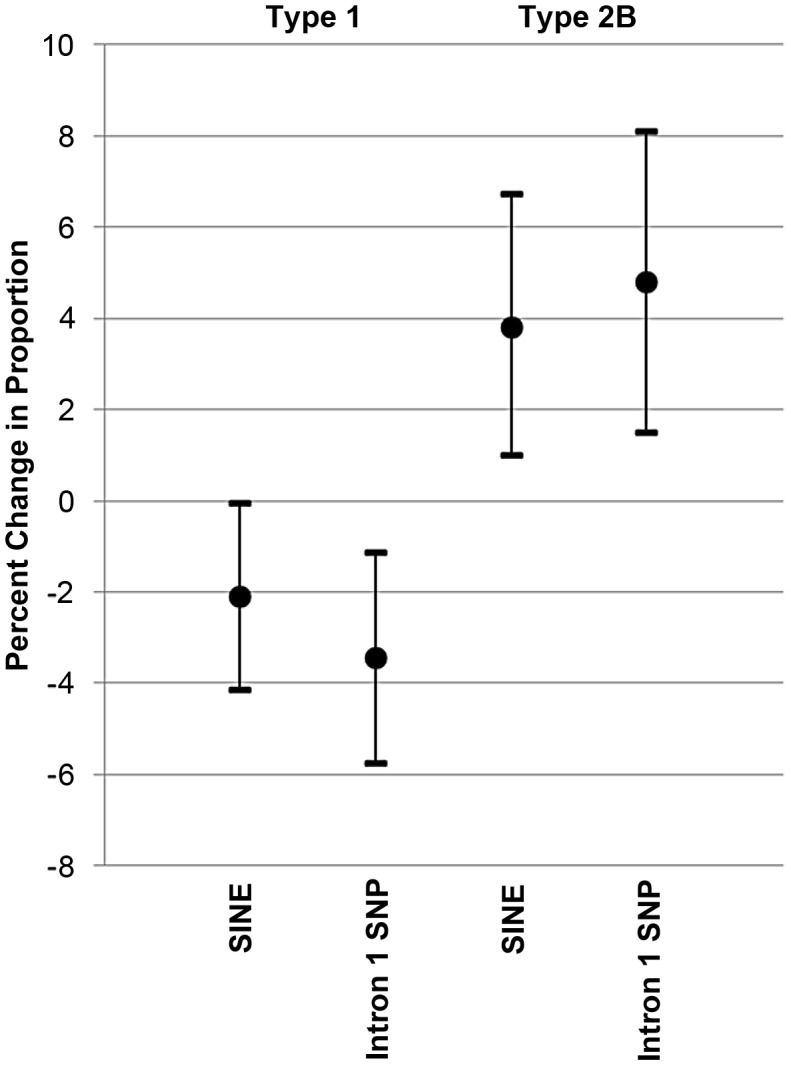
Effect of the *MSTN* SINE insertion and intron 1 “C” allele on fiber type proportions. Mean additive effect and 95% confidence intervals of the *MSTN* SINE insertion or “C” allele of the intron 1 SNP on Type 1 and Type 2B fiber type proportions.

MANOVA showed a significant association of the SINE with muscle fiber type proportions (p = 0.039) (Figure 4). The directionality and magnitude of the response was evaluated by multiple linear regression, which demonstrated that the presence of the SINE resulted in a higher proportion of Type 2B fibers (p = 0.012) and lower proportion of Type 1 fibers (p = 0.045). Each copy of the SINE increased Type 2B fiber proportions by 3.79% (95% CI = 0.98 to 6.71), and decreased Type 1 fiber proportions by 2.10% (95% CI = 0.05–4.16) (Figure 5).

MANOVA was performed in a similar manner on the intron 1 SNP data and also showed a significant association of the SNP (p = 0.008) with fiber type proportions (Figure 4). The “C” allele led to a higher proportion of Type 2B fibers (p = 0.005) and a lower proportion of Type 1 fibers (p = 0.004). In the case of Type 2B fibers, each copy of the “C” allele resulted in a 4.79% increase in Type 2B fiber proportion (95% CI = 1.50–8.09) and decrease of 3.44% (95% CI = 1.13–5.75) in the proportion of Type 1 fibers (Figure 5).

Least-square means calculated from the SINE and intron 1 SNP genotypes (considering sample size and the covariates of age and sex), and corresponding significant differences between the genotypes are found in Table 2. There was no significant evidence of non-additivity of either variant.

**Table 2:** Fiber type proportion based upon *MSTN* genotype.

		Type 1	Type 2A	Type 2B
**Intron 1 SNP**	**TT**	21.4^a^	27.0^a^	51.6^a^
	**TC**	18.2^a^	26.7^a^	55.1^a^
	**CC**	14.7^b^	24.8^a^	60.6^b^
**SINE**	**NN**	20.4^a^	27.2^a^	52.5^a^
	**NS**	15.9^b^	26.3^a^	57.8^ab^
	**SS**	15.7^b^	24.0^a^	60.3^b^

Least-squares means (proportion) of each fiber type calculated based on *MSTN* SINE and intron 1 SNP genotype. Within each marker and fiber type category, values significantly different at α = 0.05 are designated with different superscripts.

Neither the SINE nor intron 1 SNP was significantly associated with muscle fiber diameter in any statistical analysis conducted.

### ECA23—Gaited Breeds

A characteristic under strong selection within particular breeds and often a breed-defining trait is the ability to perform alternate gaits, which are characterized by variations in the pattern and timing of footfall. The standard gaits of the domestic horse and wild equids include the (flat) walk, trot, canter, and gallop (Table 3). However, instead of the two-beat contralateral gait of the trot, some horses perform the pace, a two-beat ipsilateral gait. Other natural variations in movement include four-beat ambling gaits characteristic of the Tennessee Walking Horse, Peruvian Paso, Paso Fino, and others, with unique variations in rhythm between breeds (Table 3 and Table 4).

**Table 3:** Description of common and alternative gaits characteristic of horses included in this study.

Standard Gait	Footfall Pattern and Timing
Walk	left hind - left fore - right hind - right fore
Trot	(left hind/right fore) - (right hind/left fore)
Canter/Gallop*	right hind - (left hind/right fore) - left fore
	left hind - (right hind/left fore) - right fore
**Alternative Gait**	
Pace	(right hind/right fore) - (left hind/left fore)
Lateral Amble	left hind – left fore - right hind – right fore
	left hind - left fore – right hind - right fore

* in the gallop the diagonal couplet becomes uncoupled.

Footfall pattern for each of the common gaits (walk, trot, canter, gallop) as well as those observed in gaited horses in this study, which include the 2-beat pace, and 4-beat lateral amble. A dash indicates a regular time interval between movements while parentheses indicate coupled movement. Variations in the lateral amble, which are often breed-specific involve differences in the speed of the footfall pattern, distance covered per stride, extension, and animation of limb movement. The canter can have one of two footfall patterns, which differ only by which leg is chosen as the “lead” leg.

**Table 4:** Natural gait variations present in horse breeds analyzed.

Breed	Alternative Gait(s)
Icelandic	Tölt, Pace*
Peruvian Paso	Paso llano, Sombreandando
Puerto Rican Paso Fino	Paso fino, Paso corto, Paso largo
Standardbred	Pace*
Tennessee Walking Horse	Running Walk

*not all paceNatural gait variations present in breeds in the study. All alternative gaits listed with the exception of the pace are 4-beat lateral ambles.

**Figure 6:**
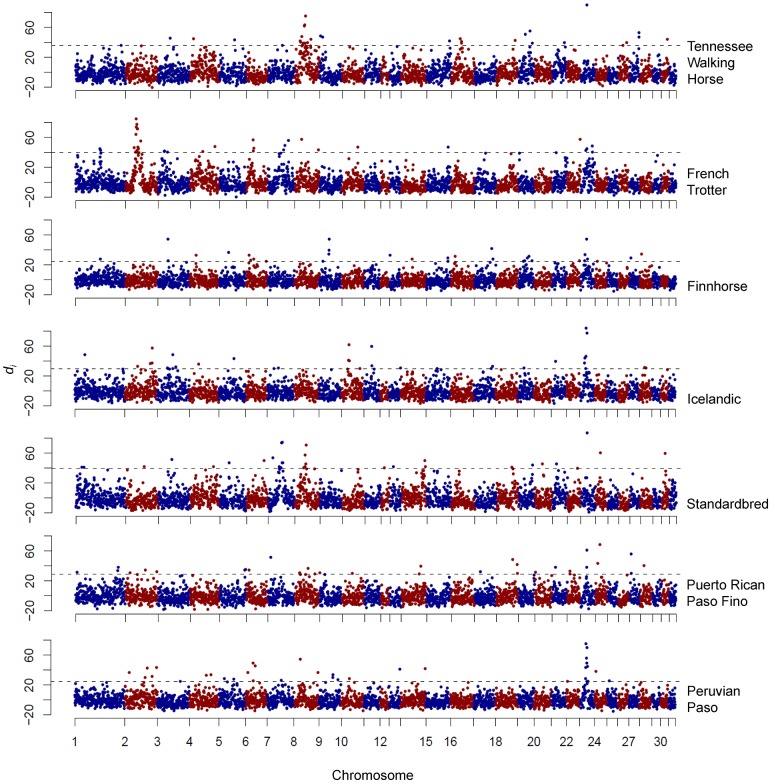
Genome-wide *d_i_* values displaying significance of ECA23 across gaited breeds. Output of the *d_i_* calculation for gaited breeds as well as those bred to race at a trot. The *d_i_* value is plotted on the y axis and each autosome is shown in the x axis in alternating colors. Each dot represents one 500 kb window. The dashed horizontal line represents the 99^th^ percentile of the empirical distribution of *d_i_* for each breed.

The window centered at 23.2 Mb on ECA23 represented the maximum *d_i_* value for four breeds: the Icelandic, Peruvian Paso, Standardbred, and Tennessee Walking Horse (Figure 6). In the Icelandic, the region of significance encompassed two consecutive, 500 kb windows, while in the Peruvian Paso, significance stretched across six consecutive windows. The Puerto Rican Paso Fino also had two adjacent, significant windows in this region although neither signified the largest *d_i_* value within the breed. This region of ECA23 was chosen for follow-up study due to the high level of significance in these breeds and the fact that all of the above breeds share the phenotype of possessing alternative gaits.

#### Haplotype analysis

**Figure 7:**
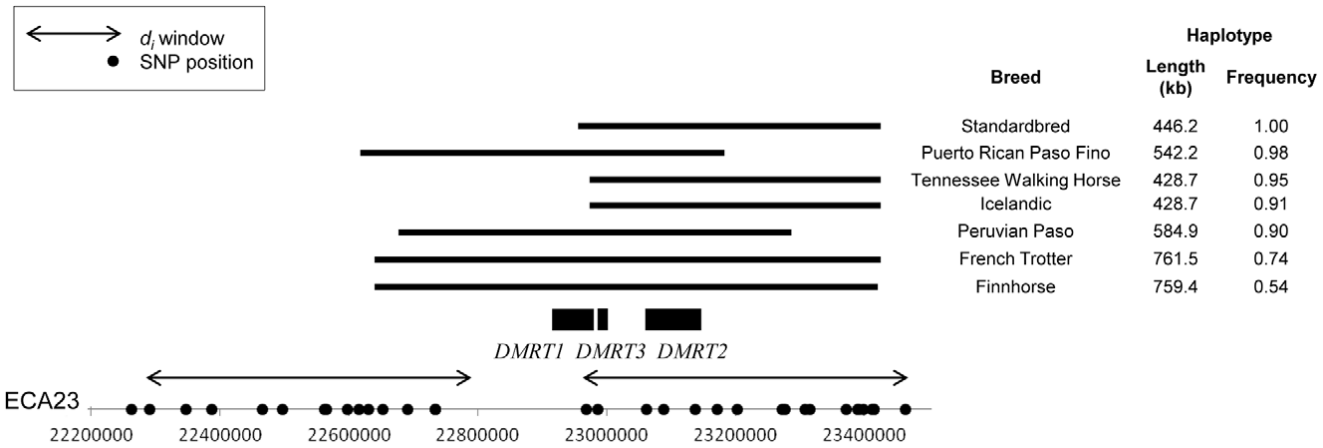
Frequency and location of extended haplotypes of gaited breeds on ECA23. ECA23 haplotype sharing in breeds that are gaited or bred to race at a trot. The common haplotype within each breed is shown as a solid, horizontal bar. *d_i_* windows are shown as double-sided arrows and SNPs used in phasing are noted as dots on the x-axis. All genes within the region shared across all breeds are noted.

Phasing of the ECA23 SNP genotypes identified haplotypes within these four breeds that ranged from 428.7 to 761.5 kb in length, and were present at high frequency (0.91 to 1.0). A haplotype just less than 186 kb was identical in the Icelandic, Peruvian Paso, Puerto Rican Paso Fino, Tennessee Walking Horse, Standardbred (non-gaited trotters), Finnhorse, and French Trotter (Figure 7). Phasing genotypes in additional Standardbred horses (N = 50), including some bred to race at the pace (gaited), found the 18 SNP, 446 kb haplotype within the breed as a whole to be fixed (data not shown).

Two genes annotated in Ecab2.0, doublesex and mab-3 related transcription factors 2 and 3 *(DMRT2, DMRT3),* are found in the ECA23 haplotype shared across gaited breeds. This “gait” haplotype was also found at low frequency in the Mangalarga Paulista (3.0%), Morgan (8.0%), and Saddlebred (30%).

### Other Potential Performance Loci

**Figure 8:**
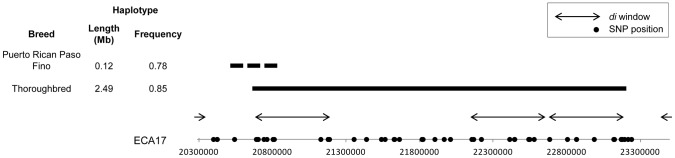
ECA17 haplotype frequency and position in the Thoroughbred and Puerto Rican Paso Fino. Position, size and frequency of extended haplotypes found under significant windows in the Puerto Rican Paso Fino and the Thoroughbred on ECA17. The haplotype in the Thoroughbred is shown as a solid, horizontal bar, while the alternate haplotype in the Puerto Rican Paso Fino is a dashed bar. Genes found within the Thoroughbred haplotype are listed in Table S2. No genes or genomic features are annotated within the haplotype found in the Puerto Rican Paso Fino.

The most significant *d_i_* value in the Thoroughbred was the first of three consecutive windows of significance on ECA17 (Figure 2). Phasing revealed a 2.49 Mb, 55 SNP haplotype in the Thoroughbred that was present in 85% of the chromosomes sampled (Figure 8). The 2.49 Mb haplotype is present but less frequent in the Hanoverian (43%), Swiss Warmblood (36%), Quarter Horse (34%), and Paint (24%). Considering all non-Thoroughbreds in the study, the haplotype is found at a frequency of 12.1% and is absent in 15 of the 33 breeds studied. This region (20.69–23.18 Mb) includes 23 annotated or predicted genes in EquCab2.0, and also includes 2 retrotransposed elements, 3 pseudogenes, and 2 known miRNA (Table S2). The Puerto Rican Paso Fino had a significant *d_i_* peak in this same region of ECA17, but had an unrelated 270 kb haplotype found in 31 of 40 chromosomes (Figure 8). No annotated genes are within the shorter haplotype found in the Puerto Rican Paso Fino. The second greatest *d_i_* value in the Thoroughbred was also highly significant in the Quarter Horse; this region on ECA14 contained a 982 kb haplotype shared across the Thoroughbred, Quarter Horse, Paint, and Swiss Warmblood, and includes ten annotated genes. This haplotype is found at a frequency of 0.33 across all 33 breeds and 0.25 when considering all but those named above (data not shown).

In other breeds, unique signals were observed on ECA2 in the French Trotter and ECA7 in the Standardbred. These putative signatures of selection span 10.4 and 12.9 Mb, respectively, and contain large, extended haplotypes across the regions of significance. A similar signature is observed in the Standardbred as well as the Tennessee Walking Horse on ECA8 where the minimum value of *d_i_* is elevated above the baseline across a large region of the chromosome (Figure 6).

### Size

Size is a phenotype easily observed and therefore selectively bred. As a result of selection, diversity in size occurs in terms of both height and mass. The extremes of size are found in the Miniature horse, which is often as small as 29 in (0.74 m) at the withers (base of the neck) and weigh less than 250 lbs (113 kg), and in the draft breeds that have a wither height of 72 in (1.83 m) or more and can weigh over 2000 lbs (907 kg).

#### ECA11

**Figure 9:**
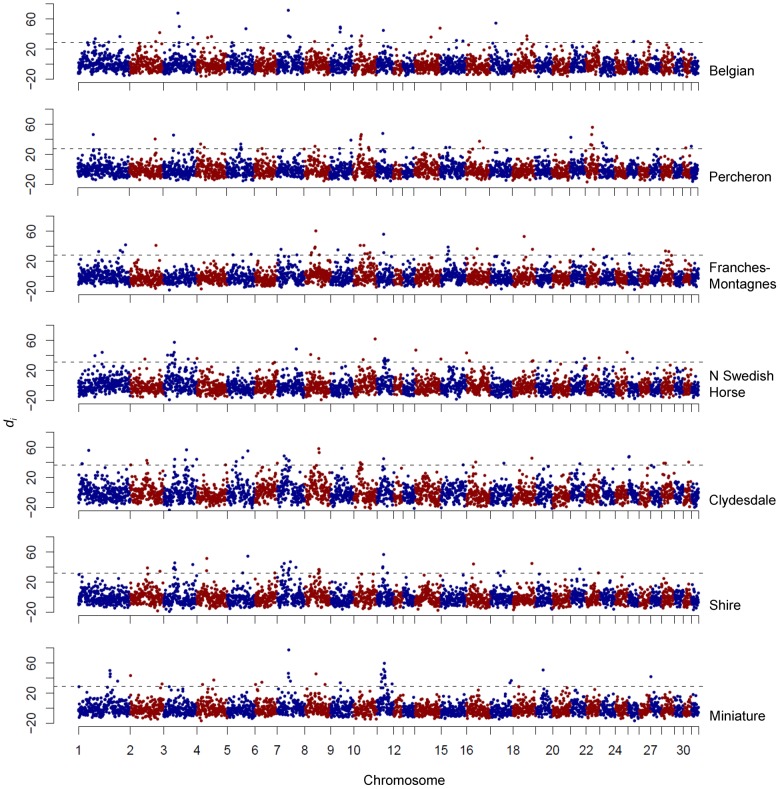
Genome-wide *d_i_* values displaying significance of ECA11 across draft breeds and the Miniature Horse. Output of the *d_i_* calculation for draft breeds and the Miniature Horse. The *d_i_* value is plotted on the y axis and each autosome is shown in the x axis in alternating colors. Each dot represents one 500 kb window. The dashed horizontal line represents the 99^th^ percentile of the empirical distribution of *d_i_* for each breed.

**Figure 10:**
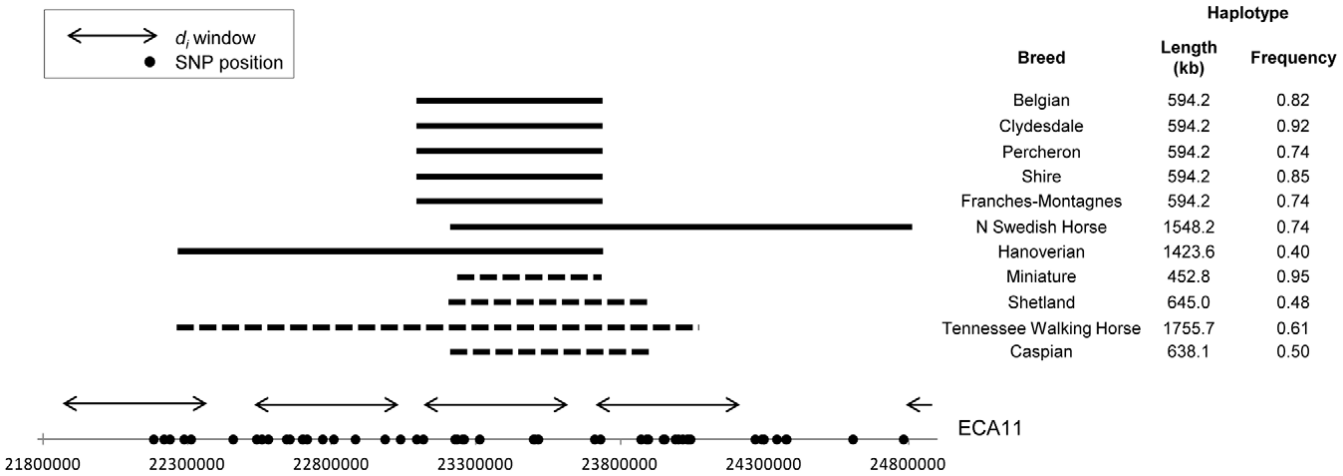
Frequency and location of extended haplotypes on ECA11 in draft breeds and the Miniature. Haplotype frequencies on ECA11 for the draft breeds, Miniature horse, Shetland and Caspian ponies, and the Tennessee Walking Horse. The common haplotype within each breed is shown as horizontal bars. The solid bars represent the haplotype similar across draft breeds while the dashed bars represent the haplotype common to the Miniature horse and related breeds. Significant *d_i_* windows are shown as double-sided arrows and SNPs used in phasing are noted as dots on the x-axis. All genes within the haplotype shared across draft breeds, including the region shared with the Miniature horse are listed in Table S2.

A region of putative selection on ECA11 was shared by all draft breeds as well as the Miniature horse and therefore hypothesized to be influential in the determination of size (Figure 9). Phasing of this region showed high conservation of haplotypes across the Belgian, Clydesdale, Franches-Montagnes, North Swedish Horse, Percheron, and Shire, ranging in frequency from 0.74 (North Swedish Horse, Franches-Montagnes) to 0.92 (Clydesdale). These 6 draft breeds share a 10 SNP, 477.3 kb haplotype centered at 23.47 Mb (Figure 10). This haplotype is found at a frequency of 0.18 in all other breeds, including 12 of 30 chromosomes in the Hanoverian, which also had a significant *d_i_* value at the same window (Figure S1).

The Miniature horse had an 8 SNP, 457.8 kb haplotype over this same region of ECA11, centered at 23.48 Mb, and conserved across 95% of the chromosomes studied. This “Miniature” haplotype is also found in moderate frequency in the Shetland pony (0.48), and the Caspian (0.50), and was common in the Tennessee Walking Horse (0.71), where it was within a longer haplotype (Figure 10). The “Miniature” haplotype overlaps 7 SNPs (452.8 kb) of the “draft” haplotype. Thirteen annotated/predicted genes and two novel snoRNA are within this overlapping region (Table S2).

#### Candidate genes for size

Apart from ECA11, *d_i_* values calculated across or adjacent to three candidate genes for size (see Methods), *IGF1*, *NCAPG,* and *HMGA2*, showed evidence of selection within this study. The Franches-Montagnes was the only breed with a significant *d_i_* value at the insulin-like growth factor 1 (*IFG1*) locus on ECA28. While a 362 kb haplotype was found in more than half of that population, it was also observed in moderate frequencies in breeds of all sizes.

**Figure 11:**
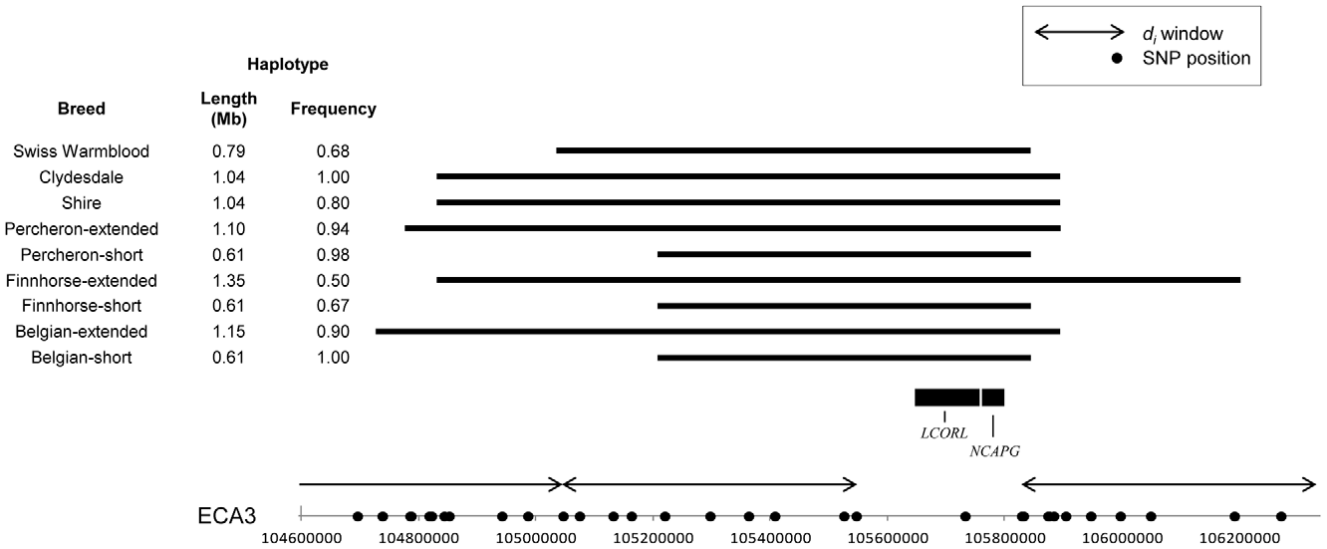
Frequency and location of extended haplotypes near *LCORL* and *NCAPG* in the draft breeds. Haplotype conservation and frequency across ECA3 near *LCORL* and *NCAPG,* candidate loci for size. SNPs on the Equine SNP50 Beadchip are designated on the x-axis as dots and windows where *d_i_* was calculated are shown with arrows. The common haplotype within each breed is shown as a solid, horizontal bar. The positions of candidate genes *LCORL* and *NCAPG* are shown; all genes found within the haplotype of interest are given in Table S2.

The candidate gene, non-SMC condensing I complex, subunit G (*NCAPG*), on ECA3 did not fall within a *d_i_* window in our data set due to low SNP density on the SNP array at that region. Yet, the window adjacent to this locus was significant in the Belgian and, upon phasing, an extended haplotype of 616.6 kb crossing *NCAPG* was found in all Belgian chromosomes; in 90% of the chromosomes the haplotype extended 1.15 Mb (Figure 11). The 616.6 kb haplotype was also fixed in the Clydesdale and common in the Percheron (0.98) and Shire (0.80). Excluding these four breeds, the ECA3 haplotype was found at a frequency of 0.096 in all other horses and had moderate frequency in the Swiss Warmblood (0.68), and Finnhorse (0.56). An extended haplotype was found surrounding a majority of the chromosomes in each of these breeds (Figure 11).

The high-mobility group AT-hook 2 gene (*HMGA2*) on ECA6 was not covered by a *d_i_* window. However, the closest window to this locus was significant in the Clydesdale and phasing found a 2.54 Mb haplotype in 91.7% of the Clydesdale chromosomes, which originated in the *d_i_* region and ended just beyond the gene. This haplotype was not frequent in other draft breeds with the exception of the Shire (52%), and is virtually absent in all other horses studied (2.0% occurrence).

## Discussion

The success of population-based statistics for detecting signatures of selection is largely dependent upon the history of the species in question and the genomic resources available. In the horse, intense, divergent selection among breeds, but shared selective pressures within breeds, as well as a whole-genome SNP genotyping platform, have led to initial successes in finding loci and polymorphisms that appear to be driving breed- and performance group-defining phenotypes. Many putative regions of selection were identified in the 33 breeds included in this study. Those pursued were chosen based upon categorization into one or more of the four criteria for prioritization of regions for investigation: those with the highest *d_i_* value within a breed, regions with at least 1 Mb of consecutive windows of significance, significant regions shared by breeds with similar phenotype, or regions showing significance across or near genes with known or suggested functional effect. This initial genome-scan led to evidence of selection for loci involved in aesthetic and performance characteristics. However, many regions left uninvestigated may also harbor important information regarding polymorphisms involved in the determination of traits of significance in these breeds.

Linkage disequilibrium generally breaks down within 400 kb in the horse genome [17]. Therefore, 500 kb windows were chosen for the calculation of the *d_i_* statistic to allow for the capture of linkage blocks as well as for the inclusion of the minimum number of SNPs (4) deemed necessary to eliminate stochastic variation and calculate a robust statistic. While formal tests for extended haplotype homozygosity (EHH) [32] were not performed, haplotype phasing of the entire SNP dataset across regions of interest was utilized to narrow and prioritize loci for follow-up work.

### Utility of the Statistic

The genetic determination of coat color in horses is largely understood [16], [18], [33]–[39] and allows for a test of the *d_i_* statistic. For example, a *d_i_* value above the threshold on ECA8 in the Norwegian Fjord is in the region to which dun coat color has been linked [18], supporting known phenotypic selection for dun coloration in this breed. However, the ability of the *d_i_* statistic to identify divergence around the known mutation for chestnut coat color in *MC1R* serves as a more appropriate and convincing demonstration of the utility of the statistic to find extended haplotypes that differ among populations. At the same time, the chestnut coat color locus, which is assumed to be contained within an extended haplotype described by [17], also highlights limitations of the statistic. The most obvious of these limitations is that due to low polymorphic SNP density and parameter settings for the calculation of *d_i_*, the *d_i_* value is not calculated for windows covering the entire genome, and in this instance was not calculated across the *MC1R* locus itself. While incomplete genome coverage can result in true signatures of selection going undetected, it can be overcome in situations where the signature of selection includes long haplotypes detectable by neighboring *d_i_* windows. This appears to be the case for *MC1R* in the Morgan population, which was fixed for an extended haplotype containing the mutant allele, allowing for detection via *d_i_* windows neighboring the locus. However, this was not the case in the Belgians, which were also fixed for the *MC1R* mutation but did not have a haplotype extending as far as that observed in the Morgan. If this were not a known mutation, this locus could have gone undetected in the Belgian. The statistic did, however, detect the *MC1R* region in the Saddlebred, which had a high proportion (0.88) of the mutant allele, and in the Finnhorse for which *MC1R* genotype data was not available but which has experienced historic selection for chestnut coat color [40]. These appear to be examples of true positive signatures of selection across this locus.

The significance of *d_i_* in the region of *MC1R* in the Shire and Exmoor breeds, which are not commonly chestnut in color, highlights another limitation of this approach: it is blinded to phenotype. While one can propose phenotype(s) that may be driving the signature of selection based upon known mutations, candidate genes found in the region, and/or by shared phenotypes among breeds sharing the same regions of significance, validation of selection and the identification of causative polymorphisms is dependent upon often labor intensive follow-up studies. In addition, false positive windows are expected as a result of genetic drift and/or founder effect, which are common phenomena in the development of domestic breeds. Shire horses are commonly bay, black, or grey, while Exmoor ponies are almost exclusively brown or dark bay; considering these two breeds, it can be hypothesized that the significant *d_i_* value at the *MC1R* locus in these breeds, and underlying haplotype, reflect selection against the chestnut coat color, or for alternative coat colors [40], [41]. However, without additional work, it cannot be determined if the *d_i_* signals in the Shire or Exmoor are false positive signals of selection, the result of genetic drift, or if the region may include other variant(s) contained within alternative haplotypes that are also under selection.

At the same window near *MC1R*, the Icelandic and Fell Pony populations had *d_i_* statistics that were slightly greater than the threshold values. The frequency of the *MC1R* haplotype found in the Morgan and Belgian was moderate in each of these breeds. Significant *d_i_* values for the Icelandic and Fell Pony could indicate that the locus is under moderate selection in these breeds or could represent false positive signals of selection resulting from the additive nature of the statistic. A similar phenomenon occurred on ECA23, the region of significance for gaited breeds. Because the statistic is additive, the extreme divergence in a few breeds in the study (*e.g.* Peruvian Paso, Tennessee Walking Horse), yielded an elevated *d_i_* values across all populations. As a result, breeds such as the Paint, Quarter Horse, Morgan, and Mongolian, still have a *d_i_* value falling into the 99^th^ percentile of the empirical distribution but with no extended, high frequency haplotype or other evidence of selection at the locus, likely representing a false positive signal of selection.

### *MSTN*

The signature of selection and underlying homozygosity in ECA18 seen in the Paint and Quarter Horse samples was profound given the relatively recent derivation of these breeds from a diverse founding stock, short blocks of linkage disequilibrium [17], and continued admixture with the Thoroughbred. The Paint and Quarter Horse do however share similar ancestry, experience continued admixture, and as a result have low genetic differentiation (JLP, unpublished data); those traits, along with shared selective pressures results in similar signals of *d_i_* in the case of ECA18 as well as across other loci.

The high frequency and size of the extended haplotype on ECA18 suggests extreme selective pressure for the phenotype that is driving this putative signature of selection. Although the haplotype was long, it was centered upon the *MSTN* gene. *MSTN* was chosen for sequencing because of its function as a negative regulator of muscle development [22]–[26], [42] and involvement in muscle fiber type determination [43]–[45], coupled with the fact that the Quarter Horse is historically known for its ability to sprint ¼ mile and continues to be selected for heavy muscling. Also, recent work has suggested that an intronic variant in *MSTN* is predictive of the best race distance for the Thoroughbred [19], [20], [46]; specifically, these studies [19], [20] suggest that horses homozygous for the “C” allele (position g.66493737C>T) are better suited for short distance racing, heterozygotes are more capable middle-distance racers, and homozygotes for the “T” allele have greater stamina for long-distance races. In addition to predicting optimal racing distance, *MSTN* has been implicated as important to racing success [47], [48] and also as having a role in body composition [49].

Fiber typing results in the Quarter Horse indicate significantly higher Type 2B and lower Type 1 gluteal muscle fiber proportions in the presence of the 5′ SINE insertion or “C” allele at the intron 1 SNP in the *MSTN* gene; this is the first histological evidence that one or both of these polymorphisms may play a functional role in muscle fiber composition in the horse. Type 1 and Type 2B muscle fibers differ in that Type 2B fibers are the fastest contracting and largest fibers in cross-sectional area, whereas Type 1 fibers are slower contracting, smaller fibers. Selection in the Quarter Horse for sprinting ability is hypothesized to favor an increase in Type 2B muscle fibers, allowing for faster and more powerful skeletal muscle contraction. Evidence of selection in the Quarter Horse for the SINE insertion and/or “C” allele of the intron 1 SNP, which are in high linkage disequilibrium in both the Quarter Horse and the Thoroughbred [19], is consistent with prior implications of the intronic “C” allele as an indicator that a Thoroughbred race horse is best suited for short distance races while the “T” allele denotes horses better suited for longer distance racing [19], [20], [47]. Although not quantified in this study, it is also possible that an increase in fiber number, in addition to the observed change in fiber proportions, may occur as a result of one or both *MSTN* mutations as observed in *MSTN* null mice [50].

The mechanism by which either *MSTN* mutation may be acting to alter muscle fiber proportions in horses is not yet understood. It has been hypothesized that the intron 1 SNP may disrupt a putative transcription factor binding site [19] and a study of a cohort of untrained, young Thoroughbreds showed increased *MSTN* skeletal muscle mRNA expression from horses homozygous for the “C” allele [51] although in that work the authors do not analyze their results in relation to the SINE insertion. A hypothesized method for a functional effect of the SINE insertion stems from its position in the promoter of the gene, effectively shifting the position of many promoter elements 227 bp upstream of the transcription start site. A displacement of the wild-type position of promoter elements, including E box, FoxO and SMAD binding sites, which have been found to be critical for regulation of *MSTN* promoter activity [52]–[55], is hypothesized to down-regulate the expression of the gene. However, further work is needed to elucidate exactly how the timing and expression of *MSTN* may change with respect to either polymorphism, and how the SINE and/or the intron 1 SNP are functionally contributing to the observed differences in muscle fiber type proportions. Finally, although the *MSTN* variants shown to be associated with muscle fiber type proportions are found most commonly within the extended haplotype putatively under selection, the haplotype derived from the SNP array is not 100% predictive of any of the *MSTN* variants we assayed; therefore, it is possible that the haplotype is tagging a different variant of selective importance that is also in linkage disequilibrium with the variants studied, or that the ascertainment of the SNPs resulting in common variants being present on the SNP chip does not allow for the detection of subtle variations in haplotype in this region.

### ECA23—Gait

In this study, we define a gaited horse as one exhibiting a different footfall pattern than displayed in the (flat) walk, trot, canter, or gallop, or a variation in the rhythm of the gait. Alternative forms of movement ranging from a 2-beat lateral gait (pace), to 4-beat diagonal and lateral ambling gaits are natural in many breeds and are often breed-defining characteristics. Alternative gaits have been selected due to increased ride comfort and for their associated visual characteristics. Horses from gaited breeds are judged upon their ability to perform the breed-specific gait and may be penalized for performing gaits not desired by the registry.

The signature of selection on ECA23 was found among all breeds in our sample that have been selected for alternative gaits as a breed-defining characteristic. The identification of one locus under a strong signal of selection shared across gaited breeds was initially surprising given the diversity of gaits found among breeds and the alternative hypothesis that each type of gait has a distinct evolutionary history. However, the significant across-breed signal of selection and conserved haplotype on ECA23 common among gaited breeds is compelling evidence that a major locus is involved in the determination of gait. Only two annotated genes are contained within the portion of this ECA23 haplotype shared across gaited breeds. Both genes are in the doublesex and mab-3 related transcription factor family (*DMRT2* and *DMRT3*). While the primary role of these genes has historically been thought to be in sex differentiation [56], [57], recent work has suggested that their role is more far-reaching [58], [59]. Concurrent with this study, a variant in *DMRT3* has been significantly associated with the ability to pace in Icelandic horses, and also appears to be necessary for horses in other breeds to perform alternate gaits [59]. The independent identification of this locus, which is contained within the haplotype shared across all gaited breeds (data not shown), supports the data suggesting that this region was targeted by selection for gait and endorses the use of this technique to identify loci under selection in the horse.

The presence of the same ECA23 haplotype in the gaited breeds within the Mangalarga Paulista, Saddlebred, and Morgan is not surprising. Certain individuals within these breeds have the ability to gait, although alternate gait is not a breed-defining characteristic. Conversely the “gait” haplotype is not found exclusively in populations that are gaited; this was also true for the *DMRT3* mutation that is presumably driving this signature of selection [59]. For example, the French Trotters and Standardbreds included in the calculation of *d_i_* do not display alternative gaits beyond the walk, trot, canter, and gallop, but are bred to race at a trot. In this study population, the “gait” haplotype also segregates (54% presence) in the Finnhorse, which is divergently selected for light draft, riding, or trotting types [40]. There is evidence that trotting performance is heritable [60] and haplotypic evidence, as well as that reported in [59], indicates that an effect of this locus on trotting aptitude cannot be ruled out.

Finally, the gaited populations in which this haplotype is found (Icelandic, Tennessee Walking Horse, Peruvian Paso, Puerto Rican Paso Fino) have various types of alternative gaits. Unless there are several variants captured within this haplotype, it appears that this locus does not itself explain the entirety of the variation in gait present in domestic horses. We therefore hypothesize that gait is a polygenic trait, and while a major locus on ECA23 may be permissive for gaitedness, variations among breeds are determined by modifying loci.

### Other Loci in Breeds Selected for Athletic Performance

The popularity and economic value of Thoroughbred racehorses have led to extreme interest in identifying genomic variants that can be utilized to predict and/or improve performance. Several previous studies have focused upon identifying genomic regions and examining candidate genes that may be associated with performance traits in this breed [15], [61], [62]. In this study, the most significant *d_i_* windows led to the identification of a large, 2.49 Mb conserved haplotype on ECA17 present in a large majority of the Thoroughbred chromosomes. All other breeds in which this haplotype is common are closely related to the Thoroughbred (JLP, unpublished data). This region of ECA17 was previously implicated as having selective importance in the Thoroughbred [15], and the syntenic region of the canine genome was noted as a region of selection in several dog breeds [9]. With long blocks of LD in the Thoroughbred breed, and many annotated and predicted genes, this region represents an area that is of further interest for evaluation via resequencing. Finally, the significant window on ECA14 in the Thoroughbred shared with the Quarter Horse and Swiss Warmblood contains an extended haplotype found in moderate frequency (0.32) across all breeds and also many annotated genes. This is another of many regions not investigated further in this study that are promising targets for future exploration.

### Size

Size, including both height and mass, is a highly-studied phenotype. Loci involved in the determination of size have been identified in the dog [4], [6], [9], [63]–[65], cattle [66]–[68] and humans [69]–[71], among other species. In horses, a majority of variation in skeletal measurements in can be explained by one principal component [72] and four loci have been identified that account for a large proportion of variance in size across breeds [73]. In addition, two significant quantitative trait loci for size have been identified in the Franches-Montagnes [74].

Although all are known for their size and strength, each draft horse population has its own, unique history. While the British Isles native Clydesdale and Shire, and mainland European Belgian and Percheron, are all considered heavy draft horses, several breeds with smaller stature but still bred for substance and strength (light draft) are included in the dataset. Therefore, a conserved haplotype across all heavy and light draft breeds, regardless of their origin, is strong evidence that the locus on ECA11 may be involved in the determination of size, as defined by height and/or mass. Support for this assertion is also found in an alternate conserved haplotype present across the same SNPs in the Miniature horse and Shetland pony and by the recent work of [73] who propose LIM and SH3 protein 1 (*LASP1*), on ECA11 as a candidate gene for size. In addition to utility in the horse, the identification of functional polymorphism(s) in this region may lend insight into size variation in other species.

Several genes are frequently proposed as important to the determination of height or mass in mammals; we examined the regions surrounding many of these genes for significant *d_i_* values, finding evidence of putative selection in three instances: *IGF1, NCAPG,* and *HGMA2*. In studies of dog, mice, and humans, insulin-like growth factor 1 (*IGF1*) has been implicated to be as a major locus in size determination [6], [64], [65], [75]–[78]. In our genome scan the light draft breed, Franches-Montagnes, was the only sample with a significant *d_i_* value in the region encompassing *IGF1* (ECA28). A common haplotype in the Franches-Montagnes was identified in 68.4% of the chromosomes of that population. This haplotype, however, was found in approximately 17% of the entire sample and in moderate frequencies in breeds that vary in size such as the Miniature, Shetland, and North Swedish Horse (data not shown). While this could indicate selection for a variant, the sharing of this haplotype with horses of all sizes suggests that either this locus is not the primary determinant of size in the horse, it is a false positive, it is under selection at moderate intensities across breeds, or that there may be one or more polymorphisms within the region found in a variety of haplotypic backgrounds.

The gene *NCAPG,* or region containing this gene (often including *LCORL*), has repeatedly been associated with body size in humans and cattle [66]–[71], [77], [79]. In horses, the region of ECA3 including *NCAPG* and *LCORL* was reported to be one of four loci that explain a significant proportion of variance to size in an across-breed study [73]. Additionally, this locus was recently associated with wither height in the Franches-Montagnes, where it was found to explain over 11 percent of variance in degressed estimated breeding value for the trait [74]. In our study the Franches-Montanges did not have a notable *d_i_* value at this window, which is expected, as the segregation of this trait within the breed is what allowed for its detection under a QTL analysis framework.

As in the case of *MC1R,* the genic region of *NCAPG* itself is not covered by a *d_i_* window due to low SNP density in the region; however the most proximal window was significant in the Belgian, which had a fixed haplotype shared with the Clydesdale. The high conservation of this haplotype across these breeds, as well as its occurrence in two sport horse breeds (Swiss Warmblood and Hanoverian), the Finnhorse, and French Trotter, but rarity in light horse and pony breeds, is additional evidence that this region may be a target of selection and playing a role in size determination of horses.

Finally, candidate gene *HGMA2* has been associated with size in the horse [73] and dog [4], [9], with height in humans [69], [71], [80], [81], and also has been shown to be critical for cardiac development [82]. Equine *HMGA2* is found at the telomeric end of ECA6, without sufficient SNP coverage for calculation of *d_i_.* However, the Clydesdale showed a significant *d_i_* value for the window nearest this gene, and along with the closely related Shire (JLP, unpublished data), was the only population with a high frequency, conserved haplotype proximal to (but not crossing) the gene. While this haplotype also includes four other genes and one novel transcript, the evidence in this and other studies suggest that this region may contain a variant with significant contribution to size that has been targeted during the derivation of the Clydesdale breed.

### Conclusion

The *d_i_* test statistic is designed to detect signatures of selection that are at or near fixation within a population, but success in detecting such loci is dependent upon and limited by the classification of populations. While classifying our samples by breed is a straight forward method, depending upon the question at hand it may be more appropriate to group breeds by performance type, shared phenotypic characters, or geographic region of origin. Although signatures of selection can be detected across breeds (*i.e.* ECA11 in the draft horses), traits with selective pressures shared across many breeds may go undetected. Finally, not all signatures of selection detected contain obvious haplotypes or candidate genes, and some regions contain no annotated genes at all. The regions on ECA8 in the Tennessee Walking Horse and Standardbred, ECA7 in the Standardbred, and ECA2 in the French Trotter are all examples of significant regions with extremely long, extended haplotypes containing many annotated genes but no obvious candidate for a known phenotype (data not shown); in these cases, the elevation of the baseline of *d_i_* values may indicate the presence of large structural variants, which would decrease recombination and result in the divergence observed. In addition, some regions of interest, including the haplotype identified on ECA17 in the Puerto Rican Paso Fino, do not include any annotated genes. These regions should not go unattended, especially in light of recent work showing significant function of non-protein coding regions on regulation of gene expression [83]–[85]. Similarly, in cases where a compelling candidate gene may be present, this does not exclude flanking non-coding regions from containing the true variation under selection.

In summary, the population structure and diverse selective pressures among breeds make the modern horse an ideal model in which to identify genomic regions and variants causative of important phenotypes. While there are some caveats to this process, including that it is blinded by phenotype and the available genomic resource does not allow for complete genome coverage, it has already yielded evidence for variants of phenotypic importance. Continued pursuit of regions of significance not investigated herein shows immense promise to uncover novel functional mutations and discoveries of importance not only to the horse but the scientific community as a whole.

## Materials and Methods

### Ethics Statement

DNA extraction and genotyping was conducted using either pulled hair or blood samples from each horse. The tissues were collected under the appropriate animal care and use protocols for each participating institution. Gluteal muscle biopsies were taken in agreement with University of Minnesota IACUC protocol 1104A98793.

### Samples and Genotyping

744 horses representing 33 breeds were collected through the Equine Genetic Diversity Consortium either as hair root samples or previously collected genotypes. For hair roots, DNA was isolated using a modification of the Puregene (Qiagen) protocol for DNA purification from tissue. Modifications include the addition of 750 µl of isopropanol rather than 300, increasing the precipitation spin time to15 m and doing so at 4°C, and washing the pellet twice. Approximately 1 µg of DNA was used for SNP genotyping using the Illumina SNP50 Beadchip according to the manufacturer's protocol. All genotype calls were extracted from the raw intensity data using GenomeStudio (Illumina) with the minimum gencall (GC) score threshold of 0.15.

Horses were chosen to be unrelated at, or more recent to, the grandparent level based upon pedigree information. In the case where pedigree information was not available, genome-sharing (pi hat) values were calculated for the autosomes in PLINK [86] (–genome) after pruning for minor allele frequency of 0.01 and genotyping rate of 0.05. One of each pair of individuals within each breed having pi hat values ≥0.25 was removed from the analysis.

### *d_i_* Statistic

Breed-specific population differentiation within 500 kb windows across the 31 autosomes was calculated using the statistic introduced by [4], *d_i_*, in Perl. Only windows with a minimum of 4 SNPs were considered. For each breed, windows of significance were determined as those with *d_i_* values falling into the 99^th^ percentile of the empirical distribution.

### Candidate Genes for Size

In addition to the significant *d_i_* regions in the draft and Miniature Horse, regions housing known candidate genes for size were investigated for significant *d_i_* values in draft and pony sized horses. Candidate genes were previously identified in studies of humans, dogs, and/or cattle; these genes included: *ZBTB38, GDF5/UQCC, HHIP, TRIP11/ATXN3, LIN28B, SH3GL3/ADAMTSL3, CDK6* [87], *PTCH1, PAPPA, CABLES1* [79], *STC2* [6], *HMGA2* [4],[6],[9],[64],[87], *GPR126* [71], [79], [87], *CHCHD7/RDHE2/PLAG1* [79], [87], *IGF1*, *SMAD2* [6], [64], *NCAPG/LOCRL* [71], [73], [79], *GDF5, EFEMP1, DLEU7, SCMH1, IHH, DYM* [71], *PRKG2, JAZF1* [70].

### Phasing

The entire SNP dataset was pruned for minor allele frequency of 0.001 and genotyping rate of greater than 95%. The data from all such SNPs within genomic regions of interested identified by the *d_i_* statistic, including those eliminated from *d_i_* calculations, were phased using fastPHASE 1.2 [88]. Parameters were set to consider 25 to 45 clusters (-Kl25 –Ku45 –Ki5) and include breeds denoted as subpopulations. Conserved haplotypes were then identified within/among the breeds showing significant *d_i_* values and the frequency of the identified haplotypes calculated within and across all breeds. The haplotypes were then examined for candidate genes that may contribute to desired phenotypes of the breed(s) of interest using the annotated equine reference sequence.

### Follow-Up

#### Myostatin sequencing and variant genotyping

Primers for sequencing the entire *MSTN* gene, also known as *GDF8,* were taken from [44]. In addition, primers to cover two gaps in the gene sequence (one each in the first and second introns) not included using the primers in [20], were designed from the draft genome assembly sequence using Primer3 [89] (Table S4). The entire gene was sequenced in 8 Quarter Horses and 6 Thoroughbreds, chosen to represent the conserved haplotype as well as alternative haplotype(s). PCR protocols were as follows: 6–7 ng of DNA was amplified with 1× buffer (plus MgCl_2_ (Qiagen)), 450 µM dNTPs (Qiagen), and 0.2 µM of each primer, 1 unit of HotStart Taq polymerase (Qiagen) and enough water to yield 16 µl reactions. For MSTN 1–3, 6–8, 11, and 12, the thermalcycler profile consisted of 20 m at 94°C, followed by 35 cycles of 94°C for 30 s, 57°C for 30 s, and 72°C for 30 s, completed by a final extension time of 10 m. Primer pairs 14a and 16 were amplified using the same conditions but with an annealing temperature of 60°C. For the remaining primer pairs (MSTN4, 5, 9, 10, 13, and 14) a touchdown protocol was used in which the samples were held at 94°C for 20 m followed by 12 cycles of 94°C for 30 s, 68°C for 30 s, decreasing by 0.5°C/cycle, and 72°C for 30 s. These cycles were followed by an additional 23 cycles at an annealing temperature of 62°C and a final extension of 10 m. PCR products were checked for amplification on a 2% agarose gel stained by ethidium bromide and visualized by ultraviolet light. PCR product was cleaned for Sanger sequencing using the ExoSAP-IT protocol (USB Corporation). DNA sequencing in both directions was carried out at the University of Minnesota's Biomedical Genomic Center.

DNA from 389 horses (Quarter Horses and Paints) available from the Equine Neuromuscular Diagnostic Laboratory was genotyped for the presence/absence of the SINE insertion using primer pair MSTN_13 [20]. Gluteal muscle biopsies from 79 healthy Quarter Horses (genotyped as part of the 389 horses above) were randomly chosen for muscle fiber type analyses and also for the intron 1 SNP (g.66493737C>T) [20]. A subset of the samples was genotyped for the promoter SNP (g.66495715T/C) [21] using primer pair MSTN14a (Table S4). Of the 389 horses above, SNP array data was available from 132 as part of this study, or for another study in the laboratory. 63 horses with SNP array data were also genotyped for the intron 1 SNP. Genotypes from these horses were then used to evaluate the association between the extended haplotype and the SINE, the haplotype and intron 1 SNP, and SINE and SNP.

#### Muscle fiber type analysis

10 µm thick sections were cut from frozen gluteal muscle tissue, preincubated at pH 4.6, and stained for myosin adenosine triphosphatase (ATPase). Muscle fiber type compositions were determined by counting the number of Type 1, 2A, and 2B fibers for a minimum of 250 fibers per biopsy and calculating a percentage composition. Maximum skeletal muscle fiber diameters were determined in 25 fibers of each type using the I solution Lite software program (IMT Technology).

A MANOVA was performed to determine significance of genotype or allele effects of the *MSTN* SINE and/or intron 1 SNP and age on muscle fiber proportions assuming an additive mode of inheritance and including sex and age at biopsy as covariates. Directionality of the effect of the SINE and intron 1 SNP was then investigated using multiple linear regression in R (http://www.r-project.org). The effect size of the SINE and intron 1 SNP was determined along with 95% confidence intervals calculated around the mean. Additionally, a genotypic model was fit and least-squared means were calculated and pairwise differences in fiber type proportion associated with genotype were tested using Fisher's least significant difference (LSD).

Fiber diameters were evaluated for association with SINE and intron 1 SNP genotype using multiple linear regression with age and sex as covariates as noted above.

### Availability of Data

All SNP genotype data is available upon request (www.animalgenome.org/repository/pub/UMN2012.1130/) for the purpose of reconstructing the analyses. The only exception is the data collected from the Tennessee Walking Horse, which, under agreement from the granting agency (to the University of Minnesota from the Foundation for the Advancement of the Tennessee Walking Show Horse (FAST) and the Tennessee Walking Horse Foundation (TWHF)), is only available under a Material Transfer Agreement (MTA) between interested individuals and the University of Minnesota.

## Supporting Information

Figure S1 Output of *d_i_* calculations for all breeds. The y axis denotes *d_i_* values while the 31 autosomes are on the x axis designated by alternating colors. Each dot represents one, 500 kb window. The dashed horizontal line represents the 99^th^ percentile of the empirical distribution of *d_i_* for each breed.
(PDF)

Table S1 Genomic coordinate (chr:bp position) of the center of the thirty-three, 500 kb windows for each breed that fell into the 99^th^ percentile of the empirical distribution and were therefore designated putative signatures of selection.
(PDF)

Table S2 Annotated genes (or other features) within high-frequency, extended haplotypes of interest.
(PDF)

Table S3 Variants (position and type) identified in sequencing *MSTN* in 6 Thoroughbred and 8 Quarter Horse individuals. The Intron 1 SNP and promoter SINE insertion used in further analyses are noted in bold. A dot (·) indicates missing data while “N” indicates no SINE insertion and “S” indicates the presence of the insertion.
(PDF)

Table S4 Primers used for sequencing of *MSTN.*
(PDF)
