# Supplementary figures and images for: Genome-Wide Analysis Reveals Selection for Important Traits in Domestic Horse Breeds
Source: PLoS Genet. 2013 Jan 17;9(1):e1003211. doi: 10.1371/journal.pgen.1003211 (PMC3547851; doi:10.1371/journal.pgen.1003211)

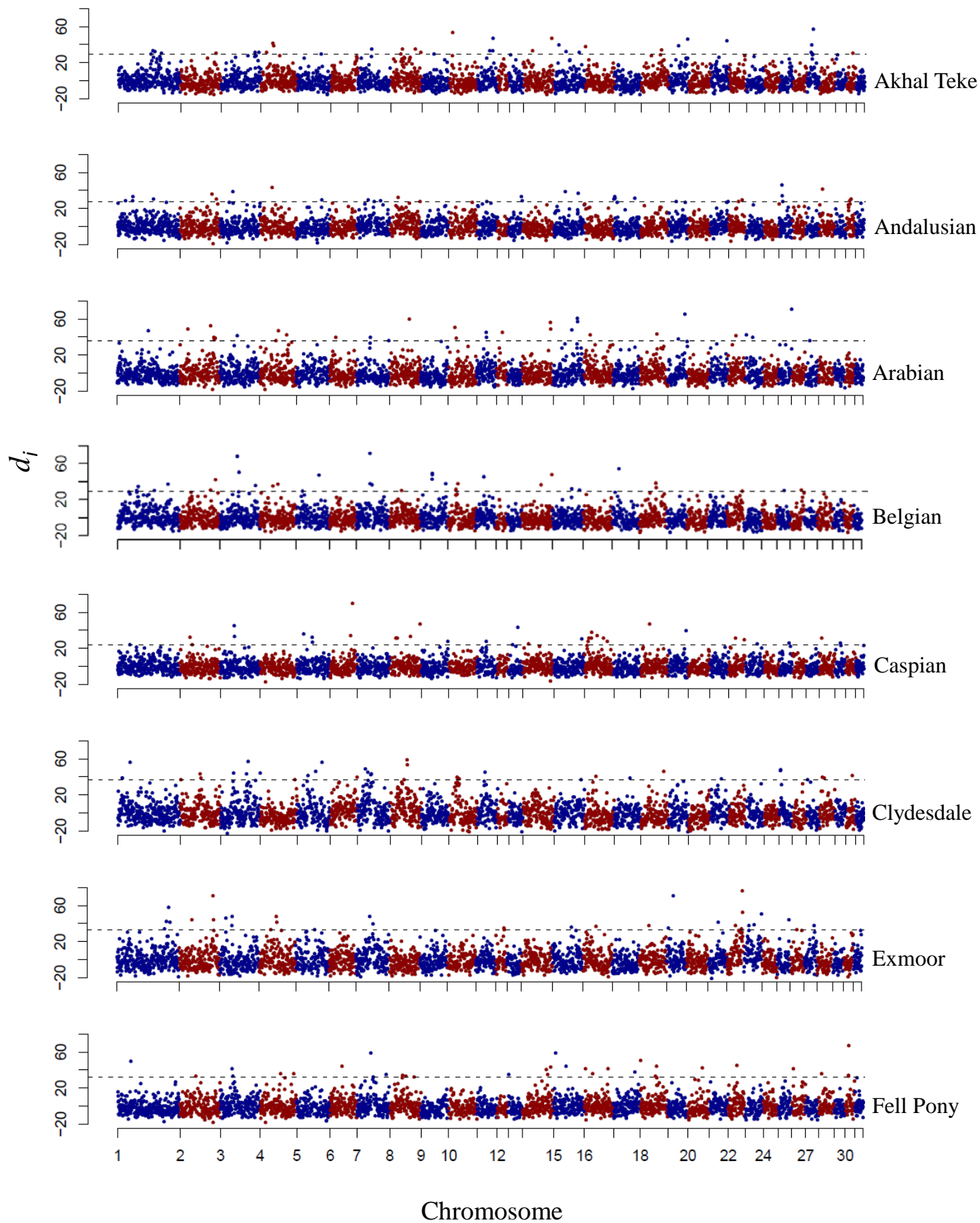

Figure S1

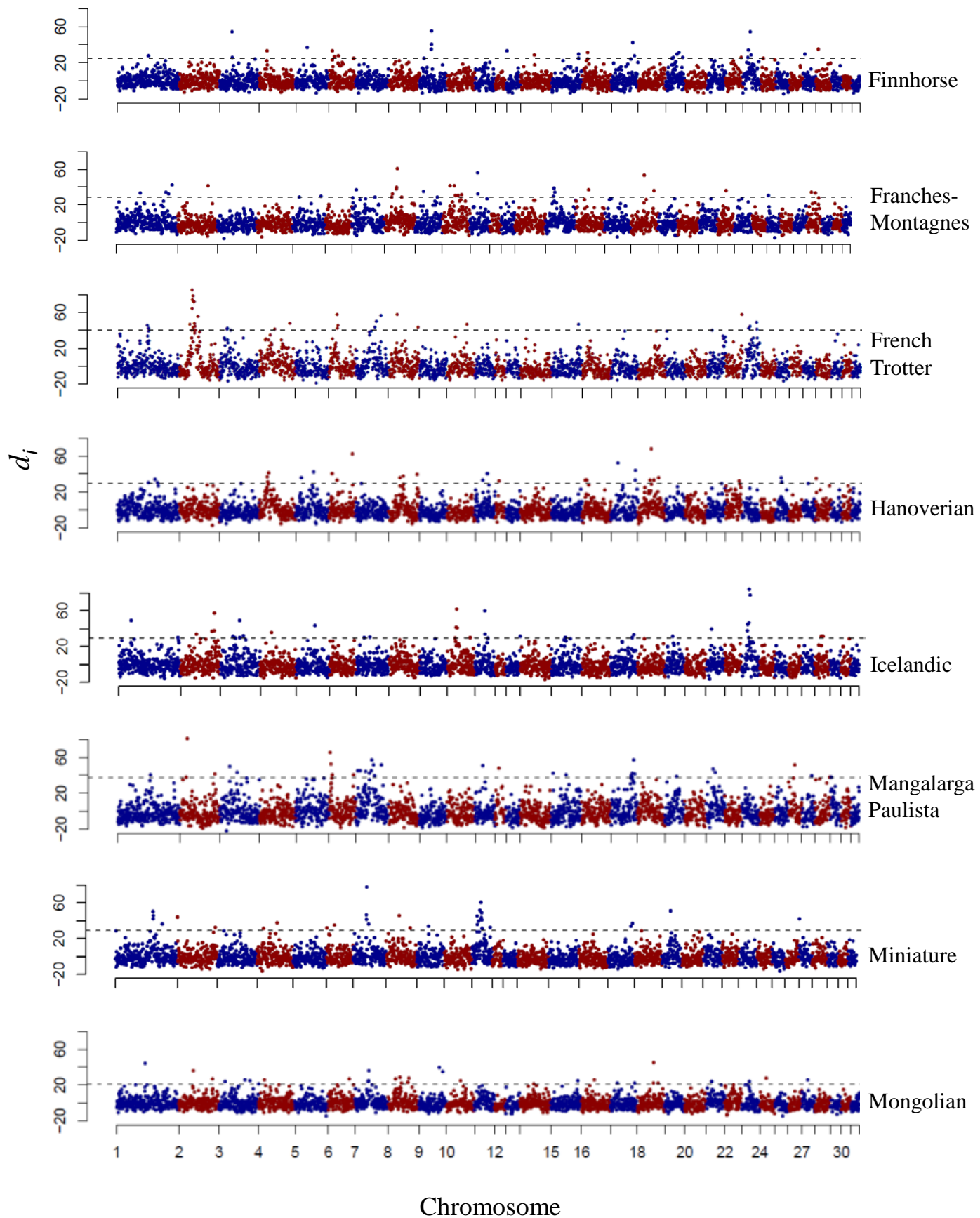

Figure S1 (cont)

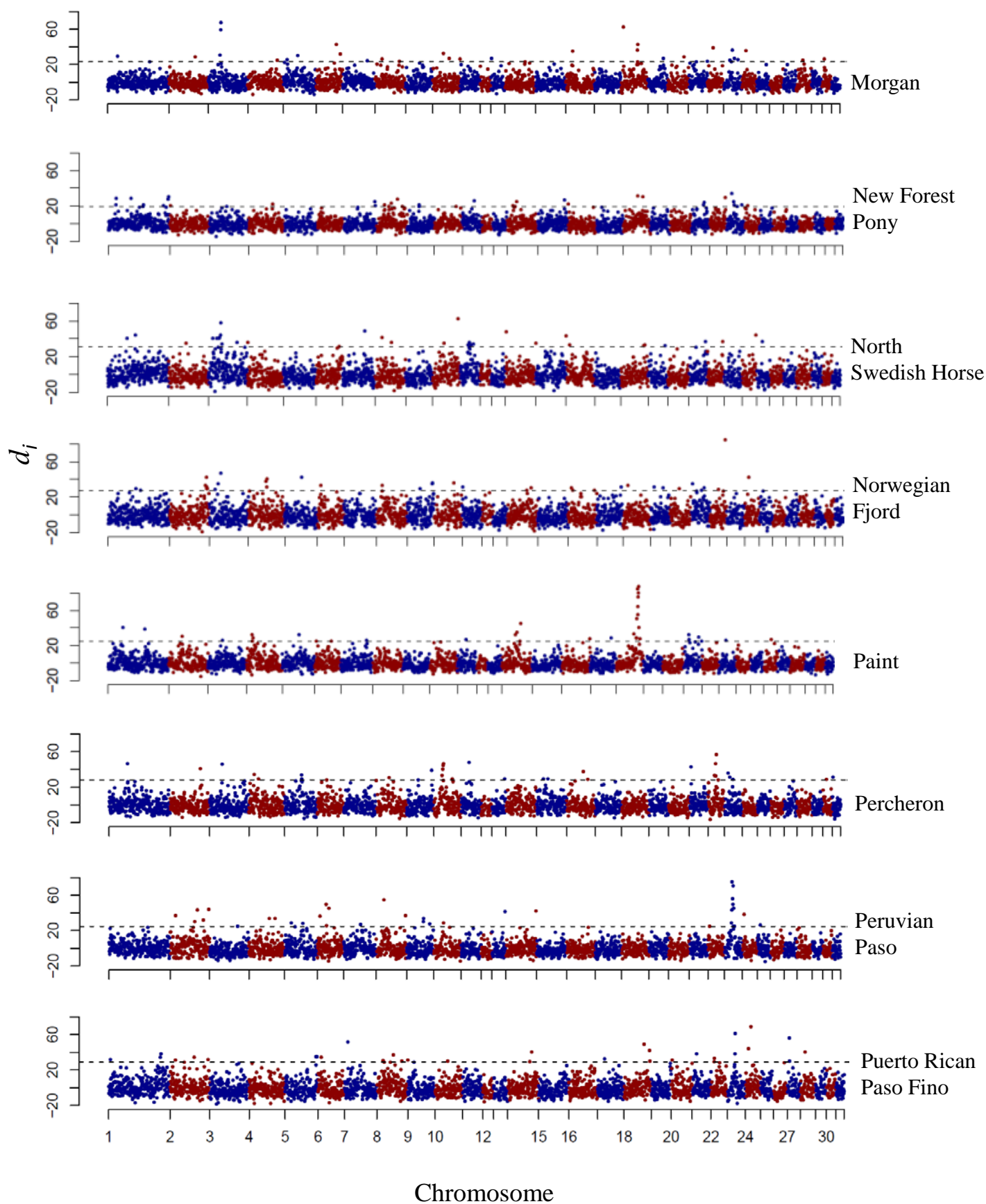

Figure S1 (cont)

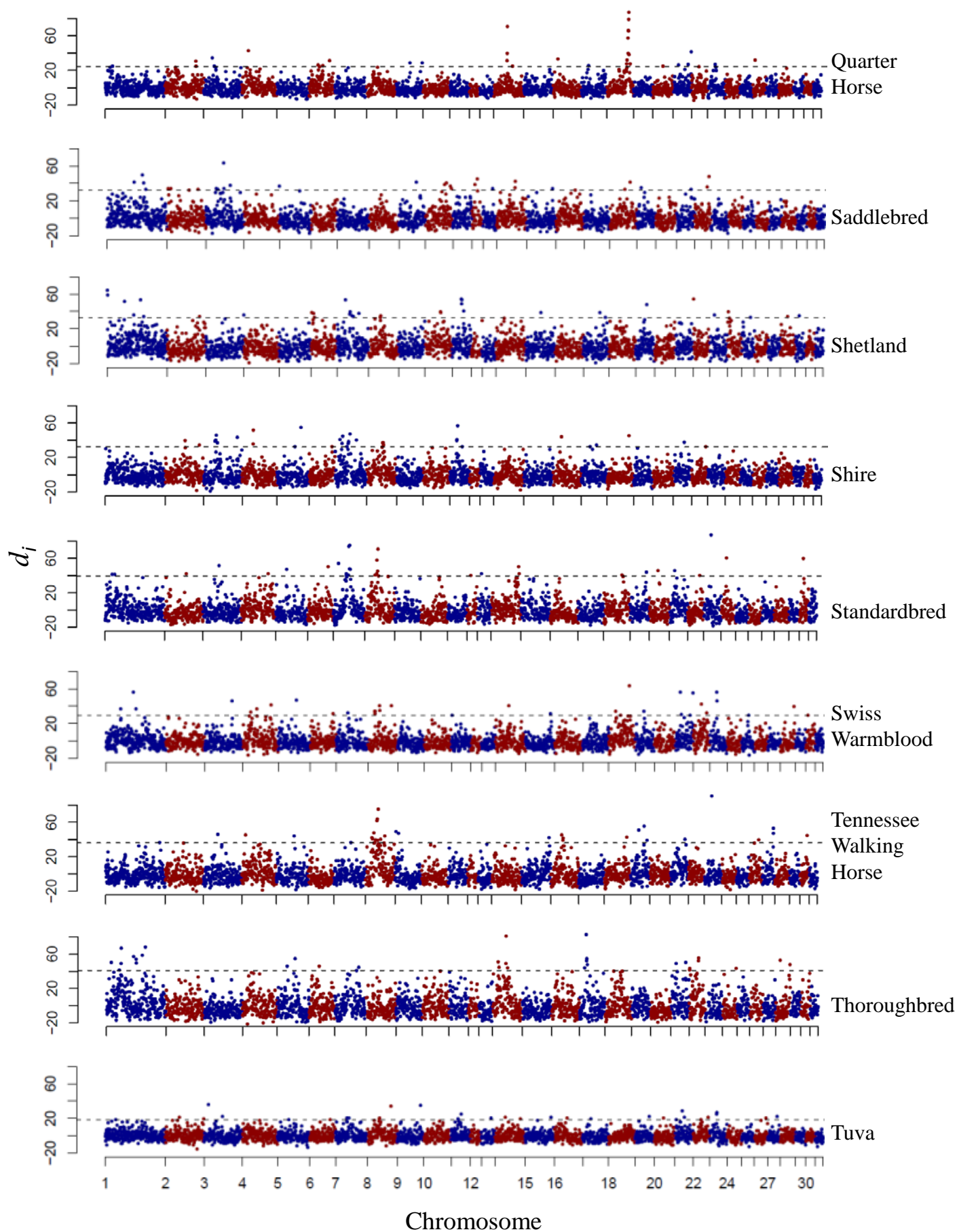

Supplement: Figure S1 — Output of di calculations for all breeds. The y axis denotes di values while the 31 autosomes are on the x axis designated by alternating colors. Each dot represents one, 500 kb window. The dashed horizontal line represents the 99th percentile of the empirical distribution of di for each breed. (PDF) [file pgen.1003211.s001.pdf]
