# Supplementary material for: Genome-Wide Analysis Reveals Selection for Important Traits in Domestic Horse Breeds
Source: PLoS Genet. 2013 Jan 17;9(1):e1003211. doi: 10.1371/journal.pgen.1003211 (PMC3547851; doi:10.1371/journal.pgen.1003211)
Supplement: Table S1 — Genomic coordinate (chr:bp position) of the center of the thirty-three, 500 kb windows for each breed that fell into the 99th percentile of the empirical distribution and were therefore designated putative signatures of selection. (PDF) [file pgen.1003211.s002.pdf]

Table S1. Genomic coordinate (chr:bp position) of the center of the thirty-three, 500 kb windows for each breed that fell into the 99th percentile of the empirical distribution and were therefore designated putative signatures of selection.

**Akhal Teke**	**Andalusian**	**Arabian**	**Belgian**	**Caspian**	**Clydesdale**	**Exmoor**	**Fell Pony**	**Finnhorse**	**Franches-Montagnes**	**French Trotter**	**Hanoverian**
1:102205365	1:23640081	1:92836057	1:56275202	2:29253849	1:12388338	1:141559392	1:39086285	1:92836057	1:72405297	1:89811759	1:92836057
1:108046292	1:44081127	2:25127188	1:61042512	3:41277613	1:13300410	1:147244029	2:47117074	3:37227339	1:149562024	1:92836057	1:112002398
1:127515479	1:45436375	2:92601193	1:109128149	3:41817820	1:36608664	1:153764548	3:34693396	3:37825015	1:157982592	2:31443015	1:119869331
2:104382151	1:106556331	2:102159442	1:149562024	5:20095922	2:58779951	1:157982592	3:37227339	4:22195106	1:168185744	2:36737292	3:63431764
3:100758100	2:94562412	2:103189105	2:91540894	5:45801283	2:61771790	2:37400190	4:60073037	5:33930869	2:91540894	2:38035768	4:23652911
3:111688033	2:108185771	2:106334223	2:106334223	5:46315160	3:38887583	2:100939871	4:99303086	6:10357668	5:20095922	2:38568800	4:24941104
4:15905004	3:37227339	3:51378517	3:51378517	6:60792087	3:76875704	2:102159442	6:35073502	6:16664747	5:85615336	2:39208463	4:27612875
4:34698424	4:34698424	4:48013130	3:55999074	6:66491461	3:81433325	3:19366645	7:40960230	6:27986271	7:13365816	2:40944330	5:17279565
4:37485250	5:73870324	4:55449773	3:105296433	8:18042600	3:117494114	3:37227339	7:48559708	6:74063729	7:69868336	2:42017895	5:53448662
7:40960230	7:33179993	4:79278889	4:38643266	8:21733196	5:32288780	3:37825015	7:86691256	9:12517775	8:24825032	2:42807353	6:9835865
8:29937334	7:51296996	6:17915950	4:52904192	8:60493821	5:55356763	4:34167967	8:38106902	9:34746009	8:36207838	2:43598118	6:24431261
8:34422420	7:69868336	7:40960230	5:67481669	8:89332508	5:73870324	4:49990964	8:48202501	9:35267151	8:37578060	2:45313577	6:70476841
8:73086390	8:24042907	7:95622851	7:40438669	9:78385433	6:79654496	4:51700499	8:72001104	9:35770488	8:39900313	2:47117074	7:17854992
8:89332508	8:89332508	8:59414786	7:40960230	11:27613048	7:24355732	7:40960230	13:2125006	13:1485739	8:52633213	2:55811541	8:35038729
10:8754201	11:28876865	10:19697977	7:46968011	13:12323238	7:31540712	7:51296996	14:71509062	14:41103285	9:25700888	3:22458450	8:44659359
11:35369931	13:38114438	10:23368490	8:36795471	13:27516469	7:40438669	12:23894923	14:75466822	15:79818862	9:67551981	3:23024232	8:86484425
11:44352630	13:40719638	11:28876865	9:34746009	14:17198854	7:42049089	12:24558133	14:84316340	15:80344415	10:23368490	4:46225658	11:23368264
11:45369127	15:33815370	11:29807882	9:35267151	15:82734531	8:50256747	15:56763560	15:6093107	16:15597151	10:36688602	4:90614058	11:37097420
14:27471476	15:71838357	12:16350337	9:35770488	16:10561202	8:51469702	16:38638668	15:37118954	17:63935482	10:39702064	6:24431261	12:11315127
14:84316340	17:405794	14:84316340	9:75629414	16:12731434	10:23368490	18:29788020	16:3794646	17:68372837	10:48168671	6:24969195	16:9829538
15:12291831	17:2593346	14:84851388	10:23878569	16:19826261	10:25915951	19:5339359	16:25260737	19:19251143	10:58572911	6:26812978	16:14435298
15:33815370	17:3804958	15:54483062	10:29745226	16:20893690	10:29745226	19:19838968	16:70616677	19:23655565	11:23368264	7:57684414	17:20940428
15:72376768	17:62200185	15:71300260	11:23368264	16:36982279	11:23368264	21:31220248	17:63935482	19:33880762	11:23962785	7:62338267	17:71982798
16:317350	19:23655565	15:71838357	14:58562422	16:55578137	15:79818862	22:25127024	18:1442565	19:39554002	15:23732407	7:75303090	17:72689827
18:59434778	21:54483816	16:18584682	14:90884366	16:67207691	16:32998762	22:43700089	18:45414883	19:50757787	15:25512766	8:26215436	18:37019276
19:28039051	22:28689582	18:50224866	15:56763560	18:26093099	17:47213263	22:45933595	18:48410000	22:45933595	15:27792702	8:88179706	18:39860166
19:55501735	22:39401137	19:31871988	15:79818862	19:52546471	18:67707777	22:46560215	20:43187121	23:15502235	16:38638668	10:58572911	18:48410000
21:48843586	25:8443219	19:50757787	17:19631092	22:19687846	21:33947822	23:15502235	22:25127024	23:17350602	18:39860166	15:79818862	18:61908546
27:12251424	25:9080525	22:22992993	18:50224866	22:45933595	25:3045502	23:31197997	26:4469593	23:23217656	18:69579598	22:47752594	22:41246919
27:14014475	25:10338312	23:4189055	18:51459337	23:34366418	25:4078681	23:53707286	28:8264904	23:25957521	22:25127024	23:19119210	25:16939693
27:16219387	28:9537681	23:23217656	22:45933595	25:29076304	28:8264904	25:34819390	30:9533798	24:7931933	25:4078681	23:23217656	25:18498415
27:18824484	30:11190107	25:38707363	25:21004886	28:6685277	28:14519521	26:19464438	30:10068990	27:6160085	28:14519521	23:42594802	27:17481989
30:19018060	30:15694650	27:12251424	26:33380395	29:16890937	30:19018060	27:29563839	30:10642643	28:6685277	28:26405894	23:43177154	28:1923754

Table S1. (continued)

Icelandic	Mangalarga Paulista	Miniature	Mongolian	Morgan	New Forest Pony	N Swedish Horse	Norwegian Fjord	Paint	Percheron	Peruvian Paso	Puerto Rican Paso Fino
1:37324605	1:97430737	1:112620338	1:84592286	1:29790268	1:22591693	1:59171841	1:83401452	1:45436375	1:55738341	2:16329197	1:5396146
2:47117074	2:19392863	1:113120360	2:43598118	2:79654345	1:23134761	1:84592286	2:108185771	1:112002398	2:91540894	2:74596559	1:155993552
2:93940340	2:22531694	1:113762369	2:100939871	3:34693396	1:68031374	2:52334217	2:111149048	2:40944330	3:37825015	2:82375270	1:157982592
2:100398489	2:105089931	1:140106731	3:18223786	3:37227339	1:106044781	3:13394607	2:111780939	3:44177551	4:15905004	2:100398489	2:16329197
2:100939871	3:29709788	2:603214	3:77717733	3:37825015	1:165827630	3:25354240	2:112375252	4:15905004	4:28633487	2:116890360	2:72613378
3:37227339	3:51378517	2:113483601	3:92196729	4:89217074	1:180236201	3:32287917	2:113483601	4:19440415	5:51188041	4:61764751	2:114717325
3:38887583	6:3459778	3:19366645	3:118774879	5:11065032	1:181412324	3:33592732	3:16977190	5:51749985	5:51749985	4:79278889	5:92753075
3:56554326	6:5615358	4:19440415	4:16838125	5:43223929	2:1529200	3:37227339	3:26513793	6:5029673	6:27986271	5:20095922	5:96105824
3:57070466	6:9835865	4:60073037	6:63433016	6:60792087	3:51378517	3:37825015	3:34121799	7:79156111	8:39900313	5:55356763	6:9335376
3:67746361	6:74063729	6:1888178	7:39895983	6:72896366	4:71237056	3:41277613	4:51700499	11:26280083	9:34746009	6:7703943	7:10116916
4:33614358	7:6533266	6:24431261	7:40438669	7:75303090	7:92929452	3:86939273	4:54252853	14:42397287	9:75629414	6:26812978	8:18620247
5:55978630	7:13365816	7:39895983	7:40960230	8:21733196	7:94492973	4:262417	4:55449773	14:47063931	10:25107856	6:27427166	8:21733196
7:24355732	7:29540215	7:40438669	8:23497931	8:74125185	8:24042907	6:69907347	5:51749985	14:58041782	10:27236368	6:35073502	8:49718815
7:40960230	7:40960230	7:40960230	8:36795471	10:32110637	8:44659359	7:67748458	6:9835865	16:85408245	10:28629007	7:51296996	8:74657508
10:26694526	7:46968011	7:46968011	8:41601829	10:50046869	8:62906066	8:21733196	8:17538265	17:63935482	10:29745226	8:24042907	8:93009642
10:28629007	7:48559708	8:40405825	8:59972319	10:82894401	9:35267151	8:50256747	9:38904743	18:53250187	10:56330822	8:89332508	10:37763499
10:29745226	7:53351032	8:73086390	8:62906066	13:1485739	11:36580369	10:33702093	9:75629414	18:60847205	11:23368264	9:50848697	14:66349082
11:28876865	7:57128127	9:34746009	9:60733866	16:20893690	14:17198854	10:76834121	9:76247079	18:61357327	13:38114438	9:51349001	14:72054197
11:29807882	7:77535387	11:15059581	9:71109944	18:7923773	14:23773621	11:23368264	10:58572911	18:61908546	15:20471150	9:69897250	17:20940428
13:42104652	11:23962785	11:15693225	10:42463983	18:50224866	14:28679683	11:27613048	11:2359201	18:64627397	15:33815370	10:29745226	18:60097420
15:42081492	12:11315127	11:19793962	14:42397287	18:51459337	15:79818862	11:32050443	14:72584360	18:65193195	16:50035128	13:39404679	18:76882666
17:63935482	15:6093107	11:23368264	15:79818862	19:46405131	16:317350	11:39893389	16:9829538	18:65815128	16:63806037	14:90884366	18:78015497
17:68938330	15:44118290	11:26280083	16:38638668	20:49599056	18:43168007	14:3628884	16:14435298	18:66328922	21:5048077	22:4388064	20:3277417
19:23122597	17:61049478	11:27025732	17:20940428	21:56830249	18:58934639	14:93241628	17:74045016	18:67069091	22:19148456	23:20907504	21:13549602
21:16741044	17:64525079	11:27613048	17:71982798	22:17408410	21:36694930	16:317350	18:13849782	18:67707777	22:22418774	23:21999458	22:9694618
23:17350602	17:68372837	11:28327356	18:48410000	23:18569445	21:38899539	16:11078185	19:11055859	18:68386361	22:22992993	23:22539658	22:10307503
23:18569445	17:71982798	11:28876865	18:49519691	23:23217656	22:45933595	18:68386361	19:38427510	18:71721918	22:25127024	23:23217656	23:22539658
23:21999458	19:35083778	11:29807882	18:61908546	23:25957521	23:16006250	18:71721918	20:27339699	21:15989330	22:30609948	23:24297889	23:23217656
23:22539658	21:21219678	11:53663674	21:16741044	23:32388178	23:21475574	19:50757787	21:4012633	21:16741044	23:11047147	23:25446201	24:9150902
23:23217656	21:27358650	17:68938330	23:11047147	23:43177154	23:23217656	21:50480673	21:38899539	21:46398408	23:16006250	23:25957521	24:16590172
23:25957521	26:20216196	17:73527194	23:23217656	24:13151827	23:32388178	22:45933595	22:25127024	21:55710684	23:25446201	23:28070897	27:6160085
28:19413076	27:29563839	19:25757994	24:19697532	28:20020863	23:45887213	24:42322495	22:45933595	23:23217656	30:11190107	24:5118684	27:6988402
28:25155125	29:3725844	27:1487636	27:17481989	30:6675329	24:9816197	25:16325925	24:13151827	26:22591756	31:1539130	25:8443219	28:14519521

Table S1. (continued)

Quarter Horse	Saddlebred	Shetland	Shire	Standardbred	Swiss Warmblood	Tennessee Walking Horse	Thoroughbred	Tuva
1:22591693	1:83401452	1:430799	2:61771790	1:21985049	1:45436375	1:168185744	1:15239917	1:32177494
2:95092072	1:109128149	1:934531	2:106334223	1:29790268	1:84592286	3:44177551	1:40272634	2:43598118
2:95766050	1:113120360	1:53461842	3:34693396	2:67726368	1:92836057	4:11876563	1:45436375	2:118788077
3:26513793	1:119869331	1:82302341	3:37227339	3:49726446	3:84366142	5:55356763	1:46081981	3:13394607
3:32287917	2:3750269	1:103110201	3:38887583	4:84044470	4:23652911	7:52824872	1:84592286	3:56554326
4:19440415	2:11658785	1:112620338	3:41817820	5:33930869	4:42087018	8:20910286	1:92836057	5:33930869
6:27427166	2:67726368	2:100939871	3:103690193	6:63956814	4:86420577	8:22290269	1:93346091	6:63433016
6:42606288	2:95766050	3:117494114	4:34167967	7:16567369	5:55978630	8:26723937	1:112002398	7:35056577
6:63433016	3:32287917	6:1888178	4:34698424	7:40438669	6:70476841	8:29937334	1:121570920	7:40960230
9:41547486	3:34121799	6:7703943	5:55356763	7:40960230	7:36761904	8:31754074	5:30850033	8:37578060
9:79549429	3:37227339	6:9835865	5:73870324	7:46968011	8:21733196	8:36207838	5:55978630	8:38106902
14:41103285	3:54594588	7:26673944	6:70476841	7:48559708	8:22290269	8:37578060	6:31481313	8:73086390
14:41872288	3:55999074	7:39895983	7:15834271	7:50631496	8:22897067	8:38106902	7:68337139	9:71109944
14:42397287	3:75485988	7:40960230	7:22015718	7:51296996	8:36207838	8:40405825	7:75303090	11:21512344
14:58041782	5:286327	7:46968011	7:24355732	7:53351032	8:37578060	8:47557951	14:17198854	11:31090202
16:14435298	9:53481490	7:51296996	7:37527086	8:32444965	8:73086390	8:60493821	14:20260124	13:30444063
17:22933951	10:58572911	7:68337139	7:40960230	8:37578060	11:1669995	9:2593081	14:41872288	14:31892985
18:60847205	10:64222554	8:38106902	7:42049089	8:38106902	14:42397287	9:10737485	14:42397287	14:78559332
18:61908546	10:76834121	10:48168671	7:46968011	8:40405825	15:77364780	15:85022973	14:47063931	15:38794959
18:64627397	10:79930255	10:48951340	7:66637525	8:41601829	18:21400451	16:33569192	17:16147103	15:79818862
18:65193195	12:8545630	11:28876865	8:50256747	8:43231982	18:37019276	16:37535285	17:20940428	16:38638668
18:65815128	12:15320581	11:29807882	8:51469702	12:11315127	18:63790592	16:38078175	17:22405099	17:25572997
18:66328922	13:30444063	11:30446632	8:52633213	13:12323238	19:26285985	18:69579598	17:22933951	19:43735342
18:67069091	14:57519927	11:36580369	11:20394412	14:85957119	21:15989330	19:25255612	17:24466631	20:28480981
18:67707777	14:58041782	15:46809952	11:20960864	14:87658235	21:19539231	19:41868862	21:15989330	21:4012633
18:68386361	15:79818862	17:51818831	11:23368264	18:56676769	21:46398408	19:50757787	21:46398408	21:22533915
18:71721918	16:59316879	19:35083778	11:37690348	19:50757787	21:55710684	21:46398408	22:1201562	21:29238431
20:31399502	18:45972798	22:411052	16:25260737	20:27339699	22:24564251	23:23217656	22:1702035	22:23557811
21:15989330	18:61908546	23:15000197	17:27194331	21:15989330	22:41246919	26:16943672	22:28689582	22:45933595
21:46398408	19:14019938	24:3772639	17:47213263	22:37215784	23:22539658	26:30412647	22:29576274	23:21999458
21:55710684	21:45756104	25:27264247	18:67707777	23:23217656	23:23217656	27:34740761	24:42322495	23:23217656
23:23217656	22:38290820	28:22207440	21:33947822	24:17240120	28:42070566	27:35244736	28:14519521	26:35617663
26:7909378	22:44209694	29:12346270	22:43700089	30:12855676	30:6675329	30:22347030	28:45009836	27:28498432
