# Supplementary material for: Genome-Wide Analysis Reveals Selection for Important Traits in Domestic Horse Breeds
Source: PLoS Genet. 2013 Jan 17;9(1):e1003211. doi: 10.1371/journal.pgen.1003211 (PMC3547851; doi:10.1371/journal.pgen.1003211)
Supplement: Table S2 — Annotated genes (or other features) within high-frequency, extended haplotypes of interest. (PDF) [file pgen.1003211.s003.pdf]

Table S2. Annotated genes (or other features) within high-frequency, extended haplotypes of interest.

Chromosome, haplotype position, and Breed(s) of interest	Gene or feature	
ECA3:105,219,978-105,832,553	LCORL	ligand dependent nuclear receptor corepressor-like
Belgian	NCAPG	non-SMC condensin I complex, subunit G
	DCAF16	DDB1 and CUL4 assocaited factor 16
	unknown	novel transcript
	novel retrotransposed element	
	novel pseudogene	
	novel snRNA	U6 splicosomal RNA
ECA11:23,118,264-23,712,507	PLXDC1	plexin domain containing 1
Draft	RPL23*	ribosomal protein L23
	FBXO47	F-box protein 47
ECA11:23,259,732-23,712,507	LASP1	LIM and SH3 protein 1
Draft and Miniature	C17orf98	chromosome 17 open reading frame 98
	CWC25	CWC25 spliceosome-associated protein homolog
	PIP4K2B	phosphatidylinositol-5-phosphate 4-kinase, type II, beta
	PSMB3*	proteasome (prosome, macropain) subunit, beta type, 3
	PCGF2	polycomb group ring finger 2
	CISD3	CDGSH iron sulfur domain 3
	MLLT6	myeloid/lymphoid or mixed-lineage leukemia
	SRCIN1	SRC kinase signaling inhibitor 1
	SOCS7	suppressor of cytokine signaling 7
	GPR179	G protein-coupled receptor 179
	unknown	novel transcript
	snoRNA (2)	
ECA17:20,690,428-23,179,269	KCNRG	potassium channel regulator
Thoroughbred	TRIM13	tripartite motif containing 13
	SPRYD7	SPRY domain containing 7
	KPNA3	karyopherin alpha 3
	EBPL	emopamil binding protein-like
	ARL11*	ADP-ribosylation factor-like 11
	RCBTB1	regulator of chromosome condensation & BTB domain containing protein 1
	PHF11	PHD finger protein 11
	SETDB2*	SET domain, bifurcated 2
	CAB39L	calcium binding protein 39-like
	CDADC1	cytidine and dCMP deaminase domain containing 1
	FNDC3A	fibronectin type III domain containing 3A
	CYSLTR2	cysteinyl leukotriene receptor 2
	RCBTB2	regulator of chromosome condensation & BTB domain containing protein 2
	RB1	retinoblastoma 1
	LPAR6*	lysophosphatidic acid receptor 6
	ITM2B	integral membrane protein 2B
	MED4	mediator complex subunit 4
	NUDT15	nudix-type motif 15
	SUCLA2*	succinate-CoA ligase, ADP-forming, beta subunit
	unknown (3)	novel transcript
	novel pseudogene (3)	
	microRNA (2)	
	retrotransposed element (2)	
ECA18:66,038,428-66,819,112	SLC40A1	solute carrier family 40, member 1
Quarter Horse and Paint	ASNSD1	asparagine synthetase domain containing 1
	ANKAR	ankyrin and armadillo repeat containing
	OSGEPL1	O-sialoglycoprotein endopeptidase-like 1
	ORMDL1	ORM1-like 1
	PMS1	postmeiotic segregation increased 1
	GDF8	growth/differentiation factor 8 precursor (myostatin)
	C2orf88	chromosome 2 open reading frame 88
	HIBCH	3-hydroxyisobutyryl-CoA hydrolase
	INPP1	inositol polyphosphate-1-phosphatase
	MFSD6	major facilitator superfamily domain containing 6
	novel pseudogene	

*gene identity predicted from orthologous sequence
