# Supplementary material for: Genome-Wide Analysis Reveals Selection for Important Traits in Domestic Horse Breeds
Source: PLoS Genet. 2013 Jan 17;9(1):e1003211. doi: 10.1371/journal.pgen.1003211 (PMC3547851; doi:10.1371/journal.pgen.1003211)
Supplement: Table S3 — Variants (position and type) identified in sequencing MSTN in 6 Thoroughbred and 8 Quarter Horse individuals. The Intron 1 SNP and promoter SINE insertion used in further analyses are noted in bold. A dot (·) indicates missing data while “N” indicates no SINE insertion and “S” indicates the presence of the insertion. (PDF) [file pgen.1003211.s004.pdf]

Table S3. Variants (position and type) identified in sequencing *MSTN* in 6 Thoroughbred and 8 Quarter Horse individuals. The Intron 1 SNP and promoter SINE insertion used in further analyses are noted in bold. A dot (·) indicates missing data while "N" indicates no SINE insertion and "S" indicates the presence of the insertion.

	Position (Ecab2.0)	66489360	66489613	66490010	66490610	66491255	66491612	66492167	66493226	66493519	66493525	66493582	**66493737**	66493775	66494218	66494302	66494362	66494367	**66495327**	66495696
	Genic Region	3' UTR	3' UTR	3'UTR	Intron 2	Intron 2	Intron 2	Intron 2	Intron 1	Intron 1	Intron 1	Intron 1	**Intron 1**	Intron 1	Intron 1	Intron 1	Intron 1	Intron 1	**Promoter**	5' UTR
	MSTN Primer Pair	1	1	2,3	4	5	6	7	9	10	10	10	**10**	10	11	11	11	12	**13,14**	14
	Reference	T	G	T	ATC	A	A	A	TCTTTT	G	T	TG	**T**	A	A	A	G	G	**NN**	A
	Variant	C	T	C	A-C	G	G	G	T----T	A	G	G	**C**	G	C	G	A	A	**SS**	G
	Variant Type	SNP	SNP	SNP	1bp del	SNP	SNP	SNP	4bp del	SNP	SNP	SNP	**SNP**	SNP	SNP	SNP	SNP	SNP	**SINE**	SNP
Horse ID	TB1	TC	GT	TT	ATC	AA	AG	AA	TCTTTT	·	·	·	·	·	AC	AA	GG	GG	NN	AA
	TB2	CC	TT	TT	ATC	AA	GG	AA	TCTTTT	GG	TT	TT	TT	AA	CC	AA	GG	GG	NN	AA
	TB3	TC	GT	TT	ATC	AA	AG	AA	TCTTTT	GG	TT	TT	TC	AA	AC	AA	GG	GG	NN	·
	TB4	·	·	TT	ATC	AA	AG	AA	TCTTTT	GG	TT	TT	TC	AA	AC	AA	GG	GG	NN	AA
	TB5	TT	GG	TT	ATC	AA	AA	AA	TCTTTT	GG	TT	TT	TC	AA	AA	AA	GG	GG	NN	AA
	TB6	CC	TT	TT	ATC	AA	GG	AA	TCTTTT	GG	TT	TT	TT	AA	CC	AA	GG	GG	NN	AA
	QH1	TT	GG	TT	ATC	AA	AA	AA	·	GG	TT	TT	CC	AA	AA	AA	GG	GG	SS	AA
	QH2	TT	GG	TT	ATC	AG	AA	AA	TCTTTT	GG	TT	TT	TC	AA	AA	AA	GG	GG	NS	·
	QH3	TT	GG	TT	A-C	AA	AA	AA	TCTTTT	GA	TT	TT	TC	AA	AA	AG	GG	GG	NS	·
	QH4	TT	GG	TT	ATC	·	AA	AA	TCTTTT	GG	TT	TT	TC	AA	AA	AA	GG	GG	NN	AA
	QH5	TT	GG	TT	ATC	AA	AA	AG	T----T	GG	TG	TG	TT	AG	AA	AA	GG	GG	NN	AG
	QH6	TC	GT	TC	ATC	AA	AG	AA	TCTTTT	GG	TT	TT	TT	AA	AC	AA	AG	GA	NN	AA
	QH7	TT	GG	TT	ATC	AA	AA	AA	·	GG	TT	TT	CC	AA	AA	AA	GG	GG	SS	·
	QH8	TT	GG	TT	ATC	AA	AA	AA	·	GG	TT	TT	CC	AA	AA	AA	GG	GG	SS	AA
