# Supplementary material for: Genome-Wide Analysis Reveals Selection for Important Traits in Domestic Horse Breeds
Source: PLoS Genet. 2013 Jan 17;9(1):e1003211. doi: 10.1371/journal.pgen.1003211 (PMC3547851; doi:10.1371/journal.pgen.1003211)
Supplement: Table S4 — Primers used for sequencing of MSTN. (PDF) [file pgen.1003211.s005.pdf]

Table S4. Primers used for sequencing of *MSTN*.

| | **Primer 1 (5'-3')** | **Primer 2 (5'-3')** | **Source$^{\omega}$** |
|---|---|---|---|
| MSTN1 | ATAAATGCAATTGTCTCAAAGTC | CCATATGCAAGTTTCCATTCC | [20] |
| MSTN2 | TCAGCCATTCAGCCTATTTG | ACGGTTGGCATTTAACCATC | [20] |
| MSTN3 | GGAGACTTGCTTTCATTTACCTG | GAAGCTTTTGGATGGGATTG | [20] |
| MSTN4 | CTCTGGGGTTTGCTTGGTG | ACCTAGGGAATGGAGGATGG | [20] |
| MSTN5 | GAAGAGGAGGGAGGGAAGAG | TTCAGTCTTCATGTGGTCTTGG | [20] |
| MSTN6 | CAAAATCAAAATAGACCTGGTACTTG | TTCATTATTTTGCAGGCTGTTG | |
| MSTN7 | AAGGTATTGTCATCTGCTTGG | CCAAGACCAGGAGAAGATGG | [20] |
| MSTN8 | GCTTGTTAGCATAGGAAACTGG | CTGAGACCCGTCAAGACTCC | [20] |
| MSTN9 | ATACCAGCGCCTGGGTTC | GACTGATTGGTACAGCTGCTCA | |
| MSTN10 | TGAAGGAATGAACTGTGGATG | GTCTGCGATCCTGCTTTACC | [20] |
| MSTN11 | TTTTGAAACTGTTGTGTCCTG | TCATAATTGCGTTTGGTTGC | [20] |
| MSTN12 | GCAAATGCTCAAATGACCTAAAC | TGTGCTGATTCTTGCTGGTC | [20] |
| MSTN13 | TGAAGATTTAGTGTTTTGTCTCC | CGAGATTCATTGTGGAGCAG | [20] |
| MSTN14 | GAGACAACTTGCCACACCAG | TGCCCTGGTAATAACAATGAAG | [20] |
| MSTN14a* | TTTAAAAATAGTTCCACATGCAAT | TGTCGTCAGGATCTATGATTGG | |
| MSTN15 | TTAGGAAGACCTGTATTAAAAGAGAAG | CAAAAGGAAAAACTGGCAAA | |
| MSTN16 | TCTTTCAGGGCATCTGGTTT | CGTAATAAAGGGGTGCCTGT | |

* Primer designed to avoid SINE insertion for genotyping of promoter variant in [21]

$^{\omega}$ If not noted, primer pair was designed for this study
